# Supplementary material for: Centrosome amplification and aneuploidy driven by the HIV-1-induced Vpr•VprBP•Plk4 complex in CD4+ T cells
Source: Nat Commun. 2024 Mar 5;15:2017. doi: 10.1038/s41467-024-46306-8 (PMC10914751; doi:10.1038/s41467-024-46306-8)
Supplement: Supplementary file 1 — Supplementary Information [file 41467_2024_46306_MOESM1_ESM.pdf]

Centrosome amplification and aneuploidy driven by the HIV-1-induced Vpr•VprBP•Plk4 complex in CD4$^+$ T cells

Jung-Eun Park[1, §], Tae-Sung Kim[1, §], Yan Zeng[1], Melissa Mikolaj[2,3], Jong Il Ahn[1], Muhammad S. Alam[4], Christina M. Monnie[5], Victoria Shi[6], Ming Zhou[7], Tae-Wook Chun[6], Frank Maldarelli[8], Kedar Narayan[2,3], Jinwoo Ahn[5], Jonathan D. Ashwell[4], Klaus Strebel[9], Kyung S. Lee[1*]

Supplementary Figures 1–8

Supplementary Tables 1–3

a

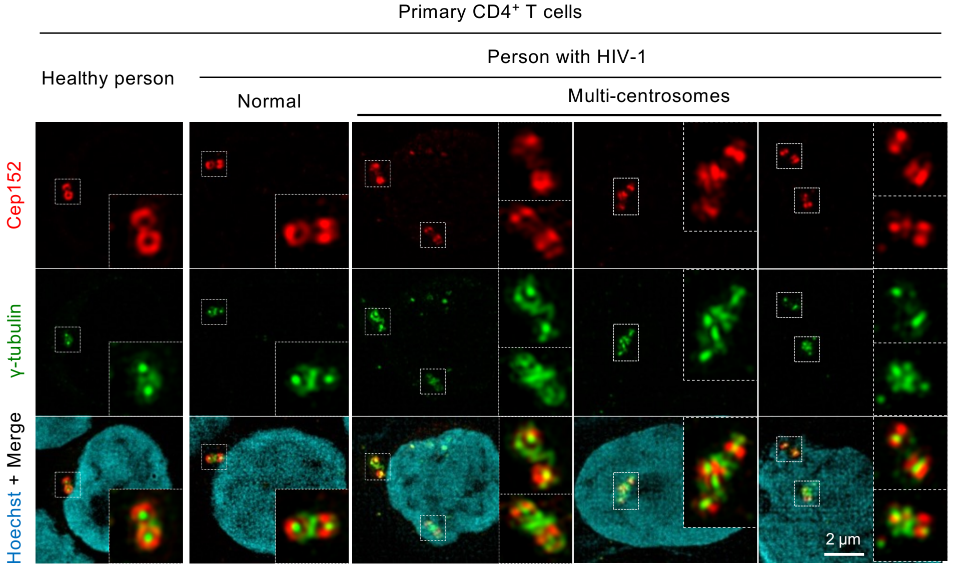

b

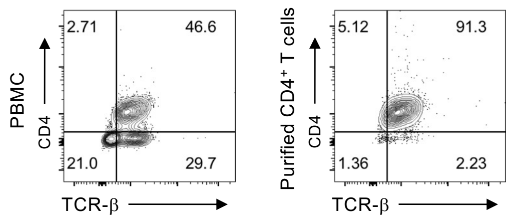

PBMCs from healthy persons

Samples	% CD4+ T cells	
	Before sorting	After sorting
#1	46.6%	91.3%
#2	28.0%	94.5%
#3	26.8%	94.3%

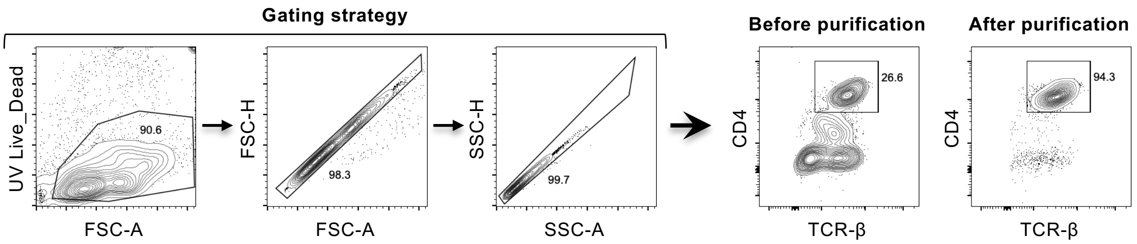

c

List of PBMC cells and centrosome counts for purified CD4+ T cells from healthy individuals

Healthy person	Sex	Age	Ethnicity	CMV status	Source	Total cells counted	%CD4+ T cells with multi-centrosomes
#1	Unknown	Unknown	Unknown	Unknown	NIH Blood Bank	523	0
#2	Unknown	Unknown	Unknown	Unknown	NIH Blood Bank	387	0
#3	M	44	White	N/A	StemCell Tech.	576	0
#4	F	56	Black	Positive	StemCell Tech.	571	0
#5	M	52	White	Negative	StemCell Tech.	778	0.13
#6	M	51	White	Negative	StemCell Tech.	754	0.13
#7	F	44	Mixed	Negative	StemCell Tech.	771	0.13
#8	F	51	Hispanic	Positive	StemCell Tech.	734	0.27
#9	M	45	White	Unknown	StemCell Tech.	561	0
#10	M	47	Native American	Negative	StemCell Tech.	508	0
#11	F	36	Hispanic	Positive	StemCell Tech.	680	0
#12	M	28	Unknown	Unknown	NIH Blood Bank	643	0
#13	F	56	White	Unknown	NIH Blood Bank	535	0
#14	F	34	Black	Unknown	NIH Blood Bank	647	0
						**Total cells = 8,668**	**Mean = 0.047**

d

List of PBMC cells obtained from people living with HIV-1 (1st set)

				Before ART					After ATR					
Patient	Sex	Age	Race/ Ethnicity	HIV-1 RNA (copies/mL)	CD4%	$CD4^+$ T cells (/µL)	Total cells counted	% $CD4^+$ T cells with multi-centrosomes	Anti-retroviral therapy (yr)	HIV-1 RNA (copies/mL)	CD4%	$CD4^+$ T cells (/µL)	Total cells counted	% $CD4^+$ T cells with multi-centrosomes
#1	M	36	Black	29599	20	264	570	**2.63**	0.7	<40	31	575	1009	**0.59**
#2	M	32	White/ Hispanic	201353	21	445	513	**3.12**	2.8	<40	36	791	1084	**1.29**
#3	F	46	White/ Hispanic	52146	22	524	579	**3.8**	0.4	<20	31	780	957	**0.63**
#4	M	21	Black	59653	19	388	508	**4.33**	1.8	<40	32	580	1013	**1.09**
#5	M	28	Black	117626	20	420	503	**2.98**	NA	NA	NA	NA	NA	NA

e

List of PBMC cells obtained from people living with HIV-1 (2nd set)

Patient	Sex	Age	Race/Ethnicity	Treatment status	HIV-1 RNA (copies/mL)	CD4%	$CD4^+$ T cells (/µL)	Total cells counted	% $CD4^+$ T cells with multi-centrosomes
#6	M	52	White	Naïve	6049	24	985	316	**5.06**
#7	M	46	Indian/ Alaska Native	Naïve	207642	32	573	330	**2.42**
#8	F	46	Hispanic	Naïve	2923	20	437	370	**5.14**
#9	M	44	White	Naïve	178923	27	475	326	**0.92**
#10	M	59	Black	During ART interruption	225658	8	155	313	**0.96**

f

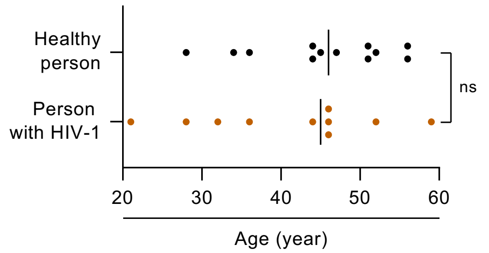

Supplementary Fig. 1. Additional examples of centrosome-over-duplicated $CD4^+$ T cells purified from the blood of people with HIV-1. a 3D-SIM images show centrosomes decorated with anti-Cep152 and anti-γ-tubulin signals. Boxes, area of enlargement. Quantified data for primary $CD4^+$ T cells with multiple centrosomes are provided in Fig. 1b. **b** Flow cytometry data showing the percentage of $CD4^+$ T cells before and after purification from PBMCs. Representative flow cytometry data are shown (top). The percentages of $CD4^+$ T cells obtained from three independently purified PBMCs are provided (top right) after analyzing them as described in the Methods. A schematic diagram illustrates FACS gating strategies used to determine the percentage of $CD4^+$ T cells before and after purification (bottom). **c–e** Clinical data of 14 healthy individuals (a) and 10 people living with HIV-1 [for the four people (#1–#4) in (**d**), paired samples were obtained before and after ART] are shown. Note that, in all four cases, ART significantly diminished the fraction of cells with multiple centrosomes, although it did not eliminate it. This appears in line with earlier observation that, while ART drastically reduces the risk of NHL and other AIDS-defining cancers, the risk of NHL in individuals with HIV-1 remains 9-fold higher than that in the general population[1]. In addition, a substantial level of Vpr still exists in the bloodstream of HIV-1 patients even after ART[2] (see Text for details). For patient #10 in (**e**), the PBMCs were obtained during the ART interruption period. **f** The age profile of 12 healthy individuals (two samples without age information were excluded) and 10 individuals with HIV-1. Bars, the median age of each group; ns, not significant.

a

Protein	Accession	Total peptide count	
		Vec	Plk4 CTD (581–970)
PLK4	J3KR82	24	365
CEP152	O94986	0	233
CEP192*	A0A0A0MR42	0	30
PCNT	O95613	1	64
CEP63	Q96MT8	0	17
CEP162	Q5TB80	0	0
CEP135	Q66GS9	0	8
PCM1	A6NNN6	0	7
CEP170*	Q5SW79	0	3
CEP78	Q5JTW2	2	4
CEP57	H0YFT8	0	4
CEP295	Q9C0D2	1	4
SPICE1	Q8N0Z3	0	3
STIL	A0A0A0MR87	0	1
CP110	O43303	0	2
CEP70	C9J710	0	1
CEP97	F8WF91	0	1
CEP112	J3QQV3	0	1
CEP250	H7C0P0	0	1
CEP290	O15078	0	1
CNTRL	Q7Z7A1	0	0
PLK1	P53350	0	0
POC5	D6RBC1	0	1
SAS6	Q6UVJ0	0	0

*The number includes 10 Cep192 fragments.

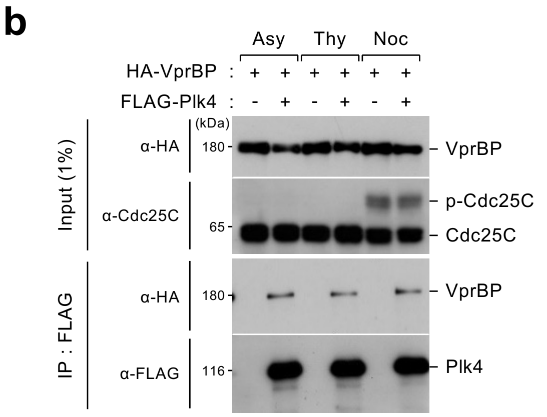

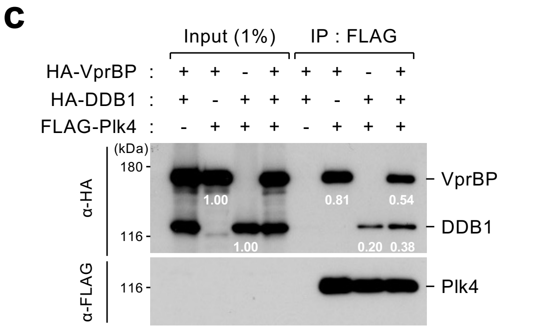

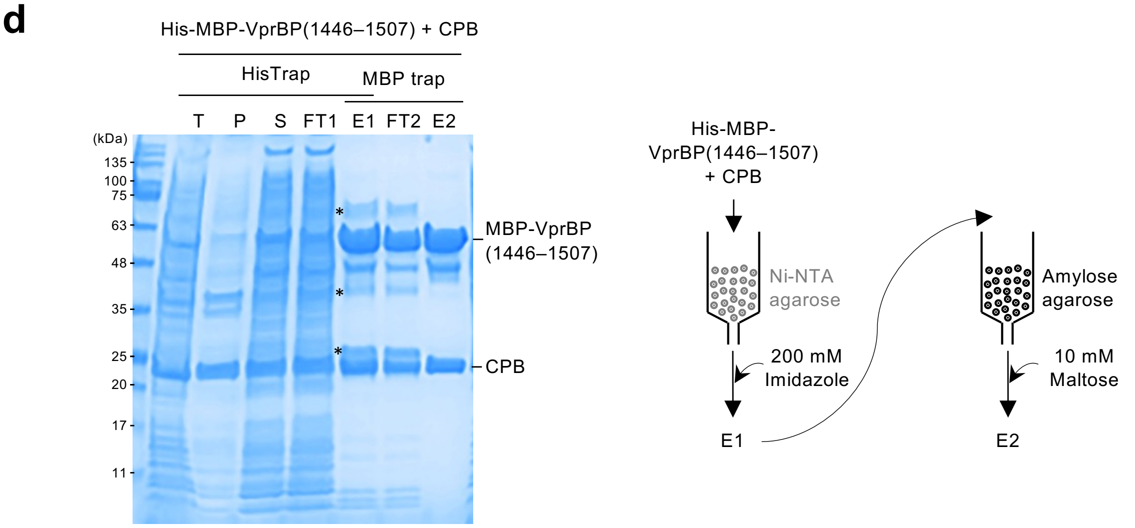

Supplementary Fig. 2. Identification of centrosomal proteins associating with Plk4 CPB and interaction between VprBP and Plk4. a Mass spectrometry data showing the centrosomal scaffold proteins copurified with the control vector or the FLAG-Plk4 CTD(581–970) ligand. The peptide counts of the listed proteins are shown. **b** Coimmunoprecipitation of VprBP with Plk4 from asynchronously growing (Asy), thymidine-treated (Thy), or nocodazole-treated (Noc) HEK293T cells transfected with the indicated constructs. p-Cdc25C, a G2/M-phase-specific hyper-phosphorylated Cdc25C. **c** Coprecipitation of VprBP and DDB1 with Plk4 immunoprecipitates from transfected HEK293T cells. Numbers, quantified signal intensities relative to the expression level of HA-VprBP or HA-DDB1. **d** In vitro interaction between VprBP(1446–1507) and CPB(581–808) using recombinant proteins. Total bacterial

(CONTINUE)

lysates expressing the indicated constructs were partially purified using His-tag affinity chromatography (HisTrap). Eluted proteins from the nickel column (E1) were then subjected to MBP-affinity chromatography, yielding the final eluate (E2) (Schematic diagram shown at right). T, total lysates; P, pellet fraction; S, supernatant fraction; FT1, HisTrap flow through; E1, HisTrap eluate subjected to MBP-affinity chromatography; FT2, MBP-trap flow through; E2, MBP-trap eluate. Asterisks, contaminating proteins.

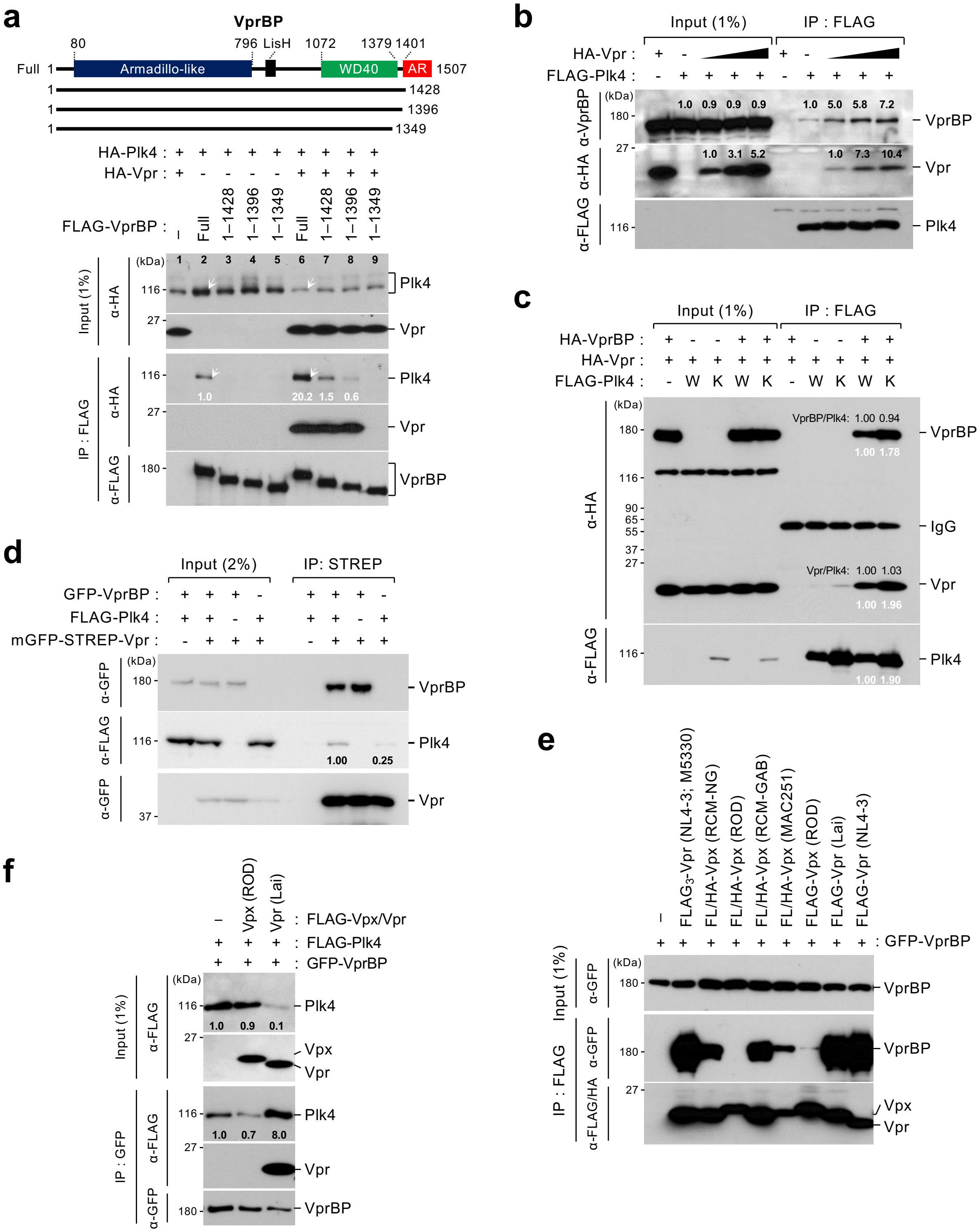
a
VprBP
80
796
LisH
1072
1379
1401
Full
1
Armadillo-like
WD40
AR
1507
1428
1396
1349
HA-Plk4 :
HA-Vpr :
FLAG-VprBP :
Full
1–1428
1–1396
1–1349
(kDa)
Input (1%)
IP : FLAG
α-HA
α-FLAG
Plk4
Vpr
VprBP
1.0
20.2
1.5
0.6
b
Input (1%)
IP : FLAG
HA-Vpr :
FLAG-Plk4 :
α-VprBP
α-HA
α-FLAG
VprBP
Vpr
Plk4
1.0 0.9 0.9 0.9
1.0 5.0 5.8 7.2
1.0 3.1 5.2
1.0 7.3 10.4
c
Input (1%)
IP : FLAG
HA-VprBP :
HA-Vpr :
FLAG-Plk4 :
W
K
VprBP/Plk4: 1.00 0.94
1.00 1.78
IgG
Vpr/Plk4: 1.00 1.03
1.00 1.96
1.00 1.90
d
Input (2%)
IP: STREP
GFP-VprBP :
FLAG-Plk4 :
mGFP-STREP-Vpr :
α-GFP
1.00
0.25
e
FLAG3-Vpr (NL4-3; M5330)
FL/HA-Vpx (RCM-NG)
FL/HA-Vpx (ROD)
FL/HA-Vpx (RCM-GAB)
FL/HA-Vpx (MAC251)
FLAG-Vpx (ROD)
FLAG-Vpr (Lai)
FLAG-Vpr (NL4-3)
: GFP-VprBP
α-FLAG/HA
Vpx
f
Vpx (ROD)
Vpr (Lai)
: FLAG-Vpx/Vpr
: FLAG-Plk4
: GFP-VprBP
IP : GFP
1.0 0.9 0.1
1.0 0.7 8.0

g

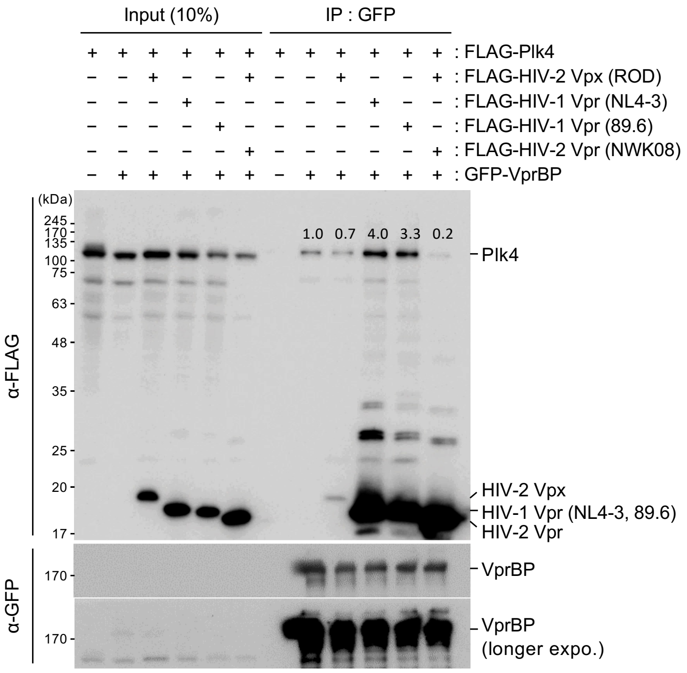
Input (10%)
IP : GFP
: FLAG-Plk4
: FLAG-HIV-2 Vpx (ROD)
: FLAG-HIV-1 Vpr (NL4-3)
: FLAG-HIV-1 Vpr (89.6)
: FLAG-HIV-2 Vpr (NWK08)
: GFP-VprBP
(kDa)
1.0 0.7 4.0 3.3 0.2
Plk4
α-FLAG
HIV-2 Vpx
HIV-1 Vpr (NL4-3, 89.6)
HIV-2 Vpr
α-GFP
VprBP
VprBP
(longer expo.)

h

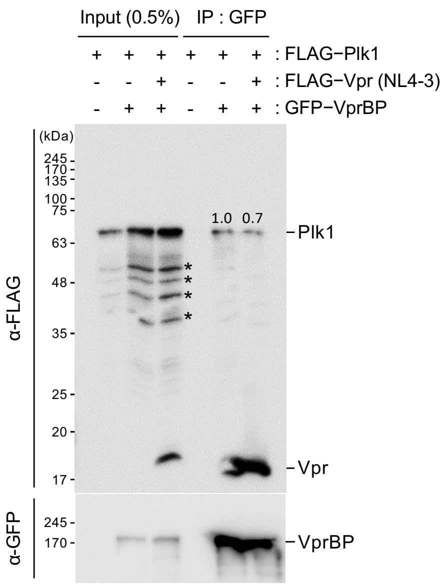
Input (0.5%)
IP : GFP
: FLAG−Plk1
: FLAG−Vpr (NL4-3)
: GFP−VprBP
(kDa)
1.0 0.7
Plk1
α-FLAG
Vpr
α-GFP
VprBP

i

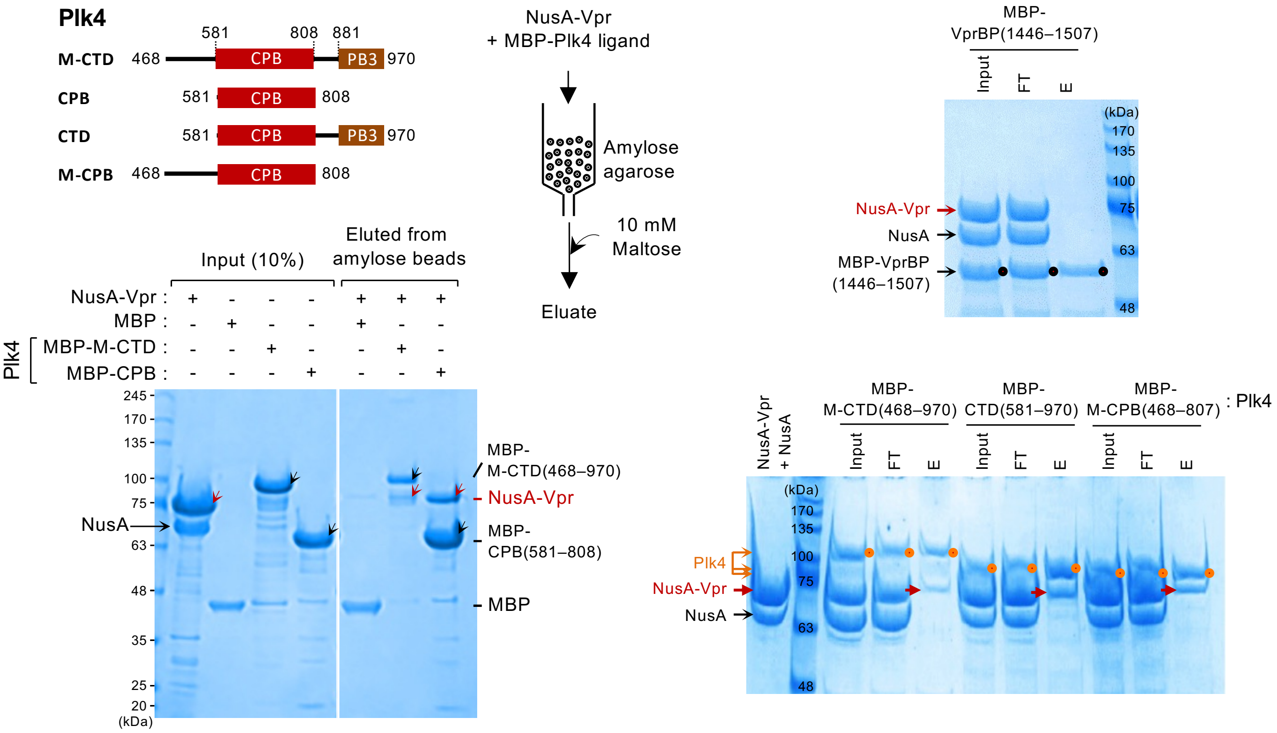
Plk4
M-CTD
CPB
CTD
M-CPB
CPB
PB3
NusA-Vpr
+ MBP-Plk4 ligand
Amylose
agarose
10 mM
Maltose
Eluate
Input (10%)
Eluted from
amylose beads
NusA-Vpr :
MBP :
MBP-M-CTD :
MBP-CPB :
NusA
MBP-
M-CTD(468–970)
NusA-Vpr
MBP-
CPB(581–808)
MBP
(kDa)
MBP-
VprBP(1446–1507)
Input
FT
E
NusA
MBP-VprBP
(1446–1507)
NusA-Vpr
+ NusA
MBP-
M-CTD(468–970)
MBP-
CTD(581–970)
MBP-
M-CPB(468–807)
: Plk4

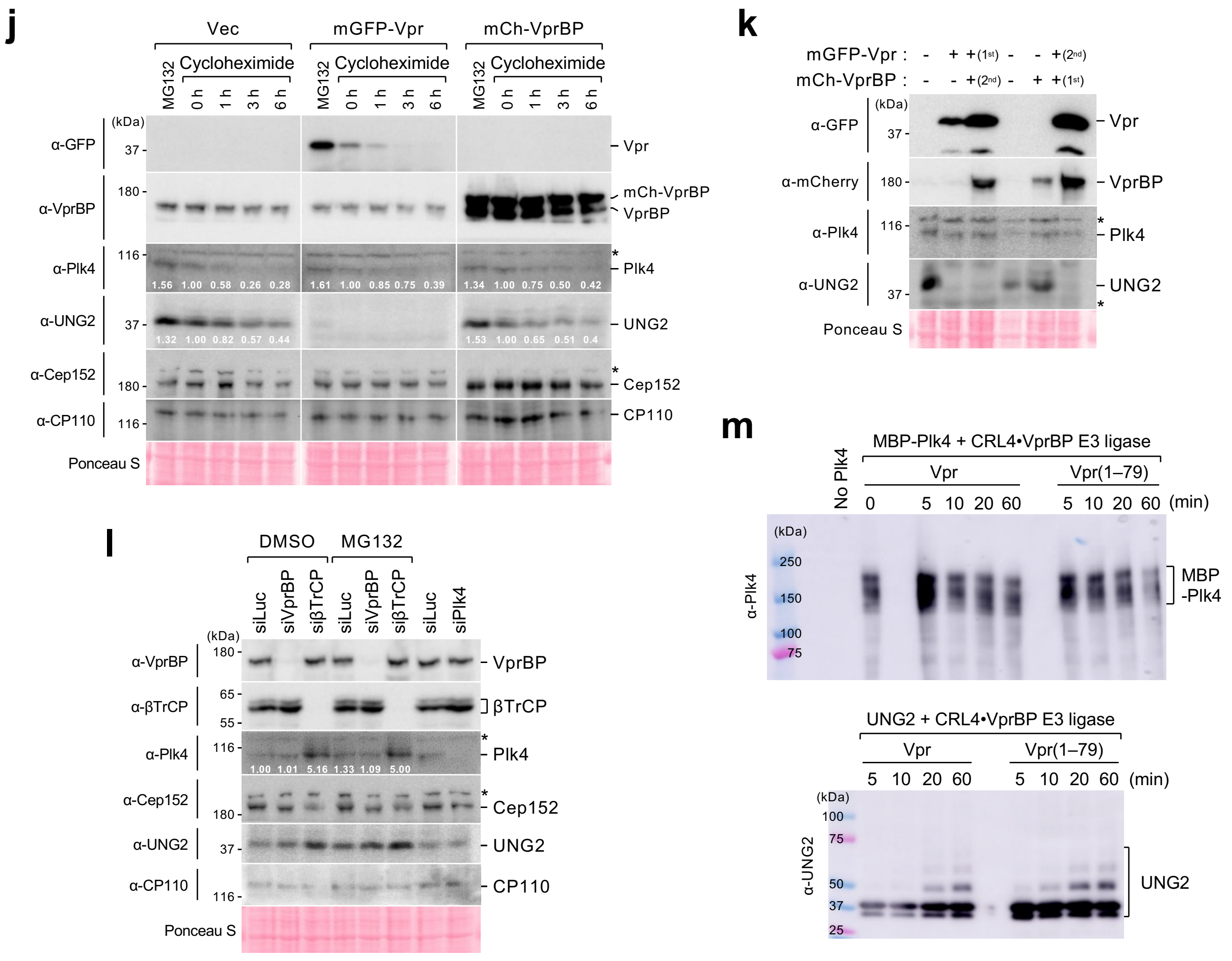

Supplementary Fig. 3. Plk4 directly interacts with Vpr independently of catalytic activity, and Plk4 is not the target of the VprBP•Vpr-mediated E3 ubiquitin ligase activity. a–h Coimmunoprecipitation analyses carried out with HEK293T cells transfected with the indicated constructs. Arrows in (**a**) denote an increased level of Plk4 bound to the full-length VprBP in the presence of Vpr (compare lane 6 to lane 2). Note that the VprBP truncates lacking the different lengths of AR (lanes 7–9) show a much-reduced or undetectable level of Plk4 binding. Black triangles in (**b**) indicate increased amounts of Vpr DNA transfected. Note that Plk4 in the input was not detected because the exposure time required for detecting the Plk4 ligand was short. The level of coprecipitating VprBP is proportional to that of Vpr expressed in the lysates. W and K in (**c**) represent Plk4 WT and the catalytically inactive Plk4 (K41M) mutant[3], respectively. An arrow in (**d**) denotes Plk4, whose binding to Vpr is enhanced in the presence of VprBP. Numbers in (**a–d** and **f–h**), relative signal intensities. Asterisks in (**h**) indicate Plk1 degradation products. The Vpr and Vpx variants in (**e–g**) are listed in Supplementary Table 1 with source information. Note that HIV-2 Vpr (Accession code: ALA65440.1) and HIV-2 Vpx (Accession code: AAB00766.1) show 54% and 28%, respectively, sequence identities with HIV-1 Vpr (NL4-3). **i** *In vitro* interaction between NusA-fused Vpr (red arrow) and Plk4 M-CTD(468–970), Plk4 CPB(581–808), Plk4 CTD(581–970), or Plk4 M-CPB(468–807) ligand (marked either arrows or dots) performed as depicted in the diagram (top left). A negative control VprBP(1446–1507) ligand (top, right; marked with black dots) was also included. Samples run by SDS-PAGE were stained with Coomassie Brilliant Blue. **j** Immunoblotting analyses for U2OS cells that stably expressed control vector, mGFP-Vpr, or mCherry-VprBP using a lentiviral system were treated with MG132 for 6 hours or cycloheximide as indicated. Numbers, quantified Plk4 and UNG2

(CONTINUE)

signals. Note that unlike UNG2, which degrades rapidly upon Vpr expression[4], the level of Plk4 remained unchanged under the same conditions. Unexpectedly, the level of CP110 remained stable, even though CP110 was suggested to be a target of the CRL4-VprBP E3 ubiquitin ligase complex[5]. Another study demonstrated that CP110 is a target of the SKP1•CUL1•Cyclin F E3 ligase complex[6]. **k** Immunoblotting analyses for U2OS cells stably expressing the indicated constructs in the order denoted. Asterisks, cross-reacting proteins. Note that, unlike UNG2, Plk4 remains stable. **l** Immunoblotting analyses for U2OS cells after silencing control luciferase, VprBP, βTrCP, or Plk4 by RNAi. Numbers, quantified Plk4 signals. Note that the level of Plk4 is increased in cells treated with siβ-TrCP, as reported previously[7], but not siVprBP. In addition, siVprBP alone does not alter the level of UNG2 because Vpr is required for triggering its degradation through the CRL4-VprBP E3 ubiquitin ligase complex[4,8]. Ponceau S-stained membranes in (**j–l**) serve as loading controls. **m** In vitro ubiquitination assays performed for bacterially expressed MBP-Plk4 or UNG2[4] using a purified CRL4 E3 ligase complex (CUL4A•RBX1•DDB1•VprBP) (see Methods for details).

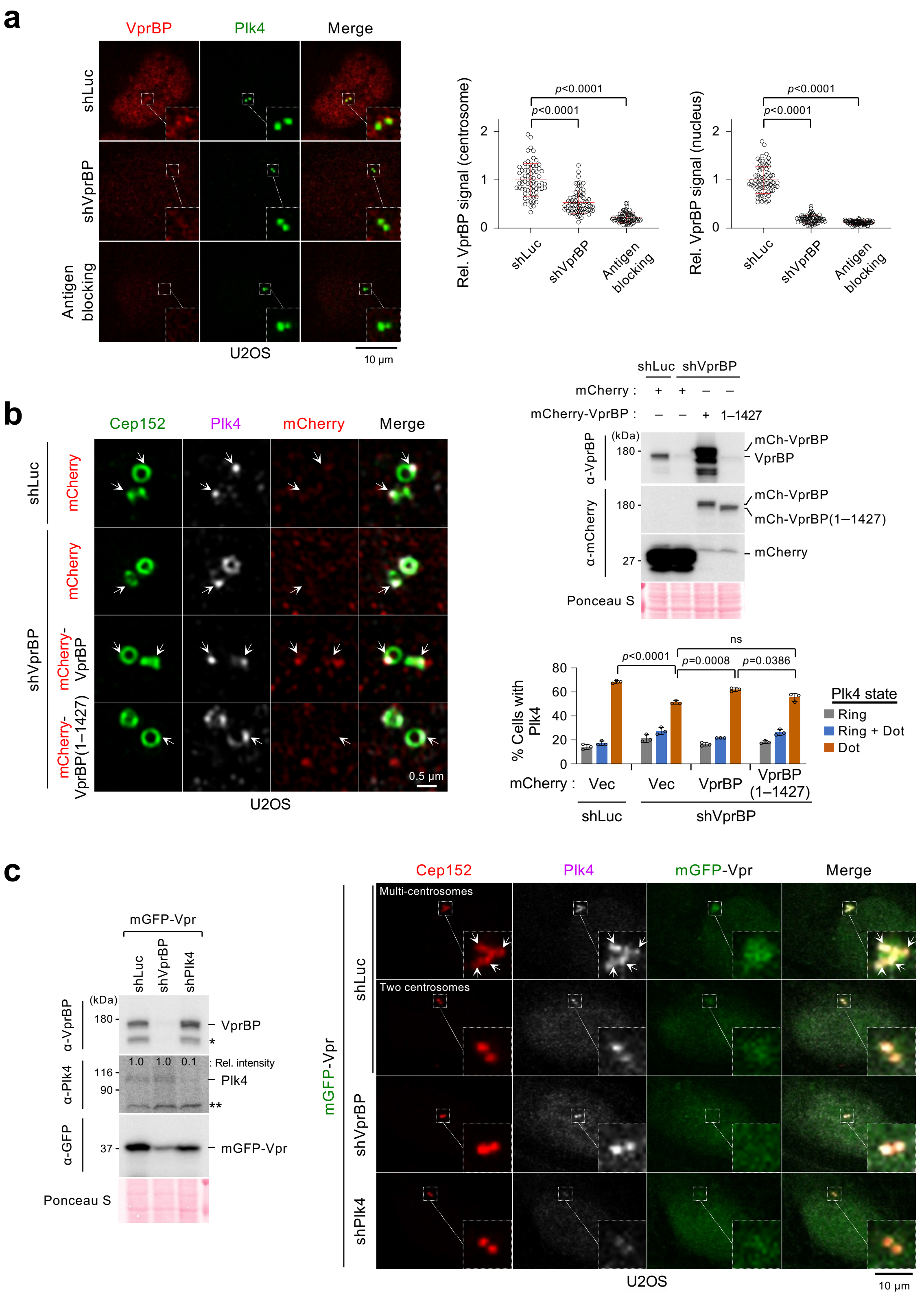
a
VprBP
Plk4
Merge
shLuc
shVprBP
Antigen blocking
U2OS
10 μm
Rel. VprBP signal (centrosome)
Rel. VprBP signal (nucleus)
p<0.0001
p<0.0001
shLuc
shVprBP
Antigen blocking
b
Cep152
Plk4
mCherry
Merge
shLuc
shVprBP
mCherry
mCherry-VprBP
mCherry-VprBP(1–1427)
0.5 μm
U2OS
shLuc shVprBP
mCherry :
mCherry-VprBP :
1–1427
(kDa)
α-VprBP
α-mCherry
180
27
mCh-VprBP
VprBP
mCh-VprBP(1–1427)
mCherry
Ponceau S
% Cells with Plk4
p<0.0001
p=0.0008
p=0.0386
ns
Plk4 state
Ring
Ring + Dot
Dot
mCherry : Vec
Vec
VprBP
VprBP (1–1427)
shLuc
shVprBP
c
mGFP-Vpr
shLuc
shVprBP
shPlk4
(kDa)
α-VprBP
α-Plk4
α-GFP
180
116
90
37
VprBP
*
1.0 1.0 0.1 : Rel. intensity
Plk4
**
mGFP-Vpr
Ponceau S
Cep152
Plk4
mGFP-Vpr
Merge
Multi-centrosomes
Two centrosomes
U2OS
10 μm

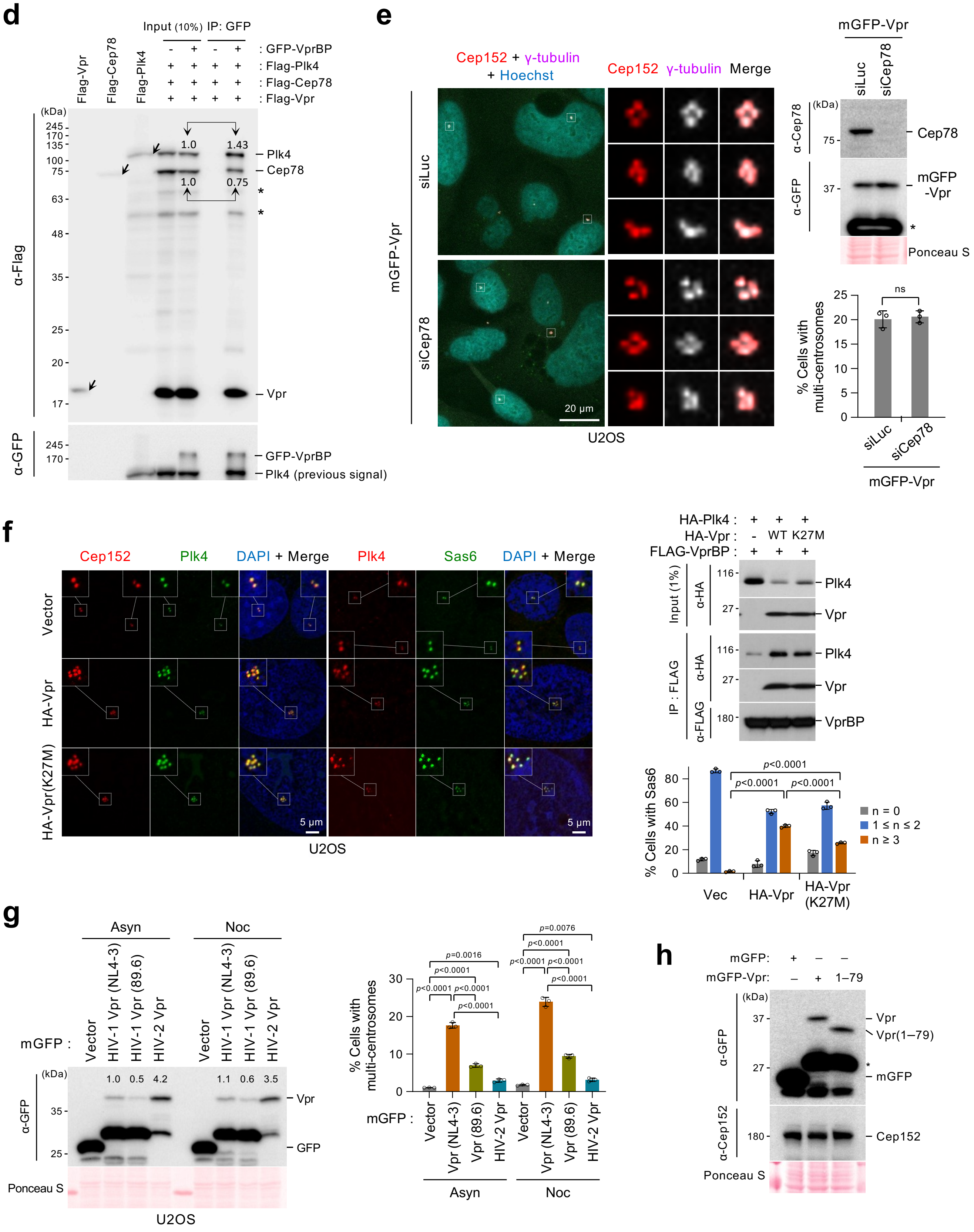
d
Input (10%) IP: GFP
: GFP-VprBP
: Flag-Plk4
: Flag-Cep78
: Flag-Vpr
Flag-Vpr
Flag-Cep78
Flag-Plk4
α-Flag
α-GFP
Plk4
Cep78
Vpr
GFP-VprBP
Plk4 (previous signal)
e
Cep152 + γ-tubulin + Hoechst
Cep152
γ-tubulin
Merge
mGFP-Vpr
siLuc
siCep78
20 μm
U2OS
α-Cep78
α-GFP
Cep78
mGFP -Vpr
Ponceau S
% Cells with multi-centrosomes
ns
f
Cep152
Plk4
DAPI + Merge
Sas6
Vector
HA-Vpr
HA-Vpr(K27M)
5 μm
U2OS
HA-Plk4 :
HA-Vpr :
FLAG-VprBP :
WT
K27M
Input (1%)
α-HA
IP : FLAG
α-FLAG
VprBP
% Cells with Sas6
p<0.0001
n = 0
1 ≤ n ≤ 2
n ≥ 3
Vec
HA-Vpr (K27M)
g
Asyn
Noc
mGFP :
Vector
HIV-1 Vpr (NL4-3)
HIV-1 Vpr (89.6)
HIV-2 Vpr
GFP
U2OS
p=0.0016
p=0.0076
Vpr (NL4-3)
Vpr (89.6)
h
mGFP:
mGFP-Vpr:
1–79
Vpr(1–79)
mGFP
α-Cep152
Cep152

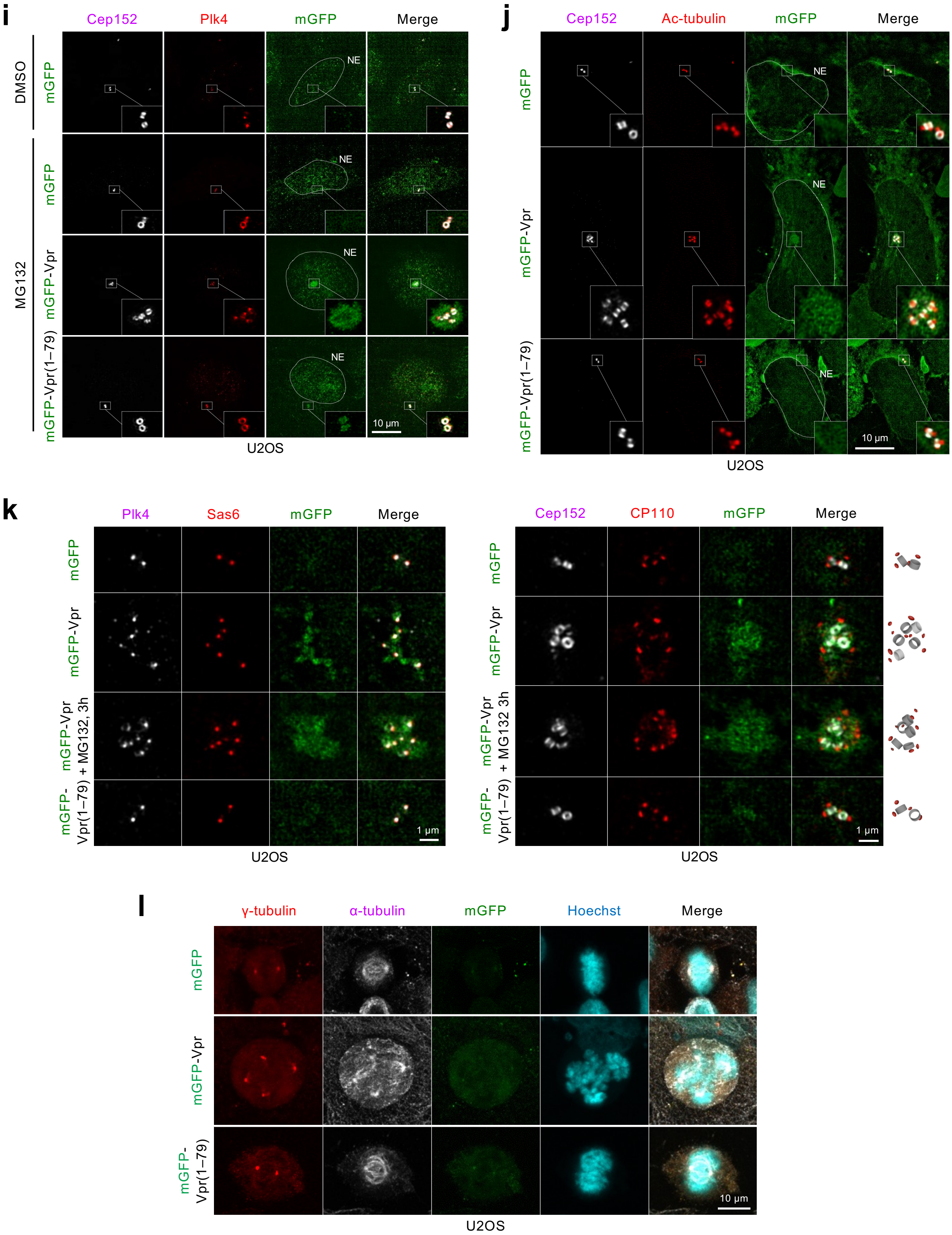
i
Cep152
Plk4
mGFP
Merge
DMSO
mGFP
MG132
mGFP
mGFP-Vpr
mGFP-Vpr(1–79)
NE
10 µm
U2OS
j
Cep152
Ac-tubulin
mGFP
Merge
mGFP
mGFP-Vpr
mGFP-Vpr(1–79)
NE
10 µm
U2OS
k
Plk4
Sas6
mGFP
Merge
mGFP
mGFP-Vpr
mGFP-Vpr
mGFP-Vpr(1–79) + MG132, 3h
1 µm
U2OS
Cep152
CP110
mGFP
Merge
mGFP
mGFP-Vpr
mGFP-Vpr
mGFP-Vpr(1–79) + MG132 3h
1 µm
U2OS
l
γ-tubulin
α-tubulin
mGFP
Hoechst
Merge
mGFP
mGFP-Vpr
mGFP-Vpr(1–79)
10 µm
U2OS

m

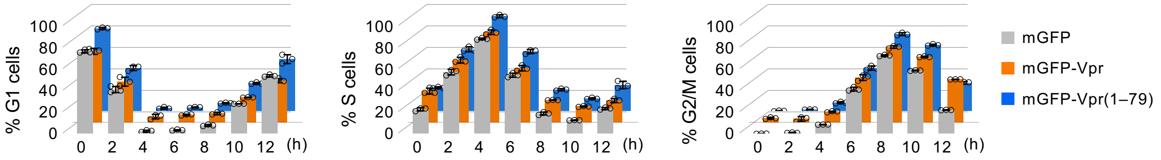

n

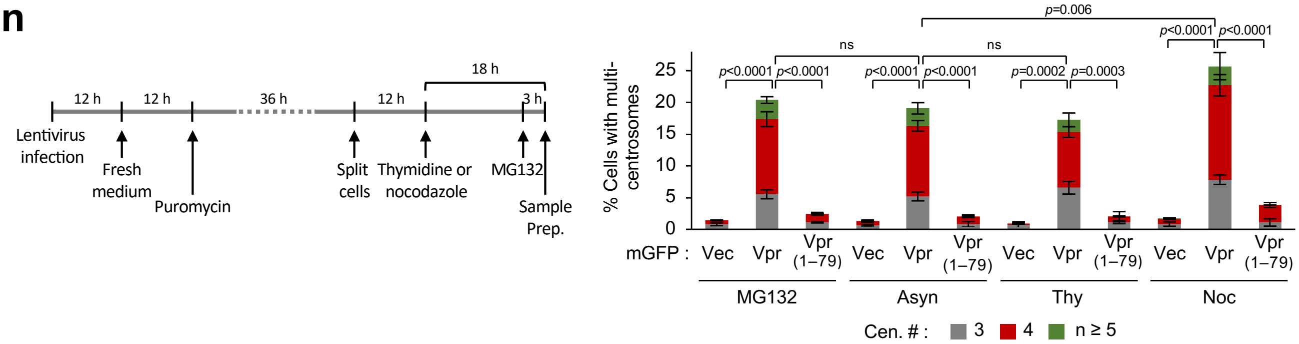

o

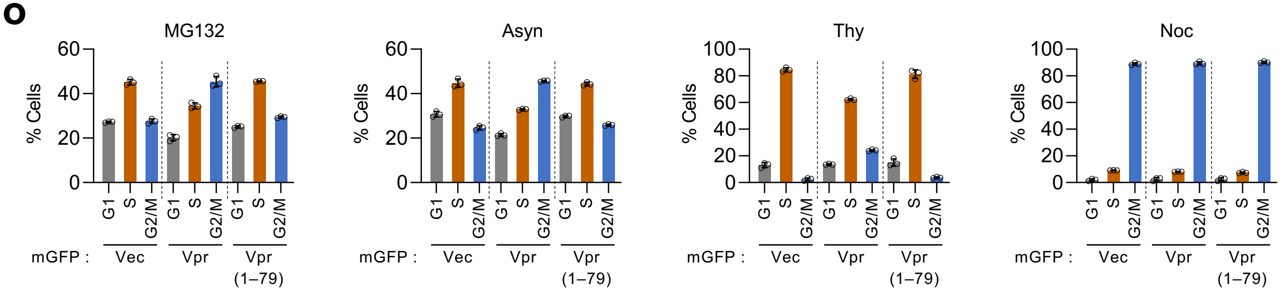

Supplementary Fig. 4. VprBP AR is required for proper Plk4 localization and Vpr induces centriole overduplication in its CT-dependent manner. a Confocal imaging and quantification of U2OS cells silenced for luciferase or VprBP and immunostained with the indicated antibodies. Boxes, areas of enlargement; antigen blocking, the sample immunostained in the presence of 5 μg/mL of VprBP(1395–1507) protein. Quantification of VprBP signal intensities was performed from three independent experiments. For centrosome-localized VprBP signals measured per experiment: n = 23 for shLuc (total n = 69); n ≥ 23 for shVprBP (total n = 70); n ≥ 22 for antigen blocking (total n = 67). For nucleus-localized VprBP signals measured per experiment: n = 24 for shLuc (total n = 72); n = 23 for shVprBP (total n = 69); n = 24 for antigen blocking (total n = 72). Bars, mean of n ± s.d.; P values, unpaired two-tailed *t*-tests. **b** 3D-SIM, immunoblotting, and quantification of U2OS cells stably expressing the indicated constructs and silenced for luciferase or VprBP using lentiviral expression and knockdown systems. Arrows, dot-state Plk4. Quantified data (graphs) were obtained from three independent experiments [per experiment, n ≥ 104 for mCherry/shLuc (total n = 317); n ≥ 100 for mCherry/shVprBP (total n = 310); n ≥ 101 for mCherry-VprBP/shVprBP (total n = 313); n ≥ 93 for mCherry-VprBP(1–1427)/shVprBP (total n = 281)]. Bars, mean of three experiments ± s.d.; P values, unpaired two-tailed *t*-tests.; ns, not significant. **c** Confocal imaging and immunoblotting of U2OS cells stably expressing mGFP-Vpr and silenced for luciferase, VprBP, or Plk4. Boxes, areas of enlargement. Asterisk, a degradation product; double asterisks, a cross-reacting protein; Numbers in (**c**), signal intensities relative to the cross-reacting (**) bands; arrows (right), multiple Plk4 and Cep152 signals observed in cells with a high level of centrosome-associated mGFP-Vpr. Quantified results are provided in Fig. 4b,c. **d** Coimmunoprecipitation and immunoblotting analyses performed using HEK293T cells transfected with indicated constructs. Arrows, target proteins in the lysates; numbers, relative signal intensities compared between inputs and their respective coimmunoprecipitates. Asterisks, degradation

(CONTINUE)

products. **e** Confocal images, immunoblots, and quantification of cells exhibiting multiple centrosomes analyzed with U2OS cells expressing mGFP-Vpr and silenced for control luciferase (siLuc) or Cep78 (siCep78). Boxes, area of enlargement. Asterisk, a degradation product. The quantified graph at the bottom right was obtained from three independent experiments [per experiment, n ≥ 446 for mGFP-Vpr/siLuc (total n = 1397); n ≥ 450 for mGFP-Vpr/siCep78 (total n = 1447)]. Bars, mean of three experiments ± s.d; ns, not significant. **f** Confocal imaging, immunoblotting, and quantification of U2OS cells transfected with the indicated constructs. Quantified data (graphs) were obtained from three independent experiments [per experiment, n ≥ 115 for vector (total n = 439); n ≥ 103 for HA-Vpr (total n = 417); n ≥ 117 for HA-Vpr(K27M) (total n = 451)]. Bars, mean of three experiments ± s.d; P values, unpaired two-tailed *t*-tests. **g** Immunoblotting analysis (left) and quantification of cells with multiple centrosomes (right) using U2OS cells stably expressing the indicated mGFP-Vpr construct. The Ponceau S-stained membrane provides loading controls (left). Quantification (right) was performed using both asynchronously growing (Asyn) and nocodazole-treated (Noc) cells to minimize the effect of cell cycle differences among samples. Quantified data are obtained from three independent experiments [per experiment, n ≥ 556 for Asyn/Vector (total n = 2023); n ≥ 551 for Asyn/Vpr(NL4-3) (total n = 1943); n ≥ 560 for Asyn/Vpr(89.6) (total n = 1745); n ≥ 646 for Asyn/HIV-2 Vpr (total n = 1989); n ≥ 501 for Noc/Vector (total n = 1606); n ≥ 545 for Noc/Vpr(NL4-3) (total n = 1692); n ≥ 514 for Noc/Vpr(89.6) (total n = 1572); n ≥ 511 for Noc/HIV-2 Vpr (total n = 1664)]. Bars, mean of three experiments ± s.d.; P values, unpaired two-tailed *t*-tests. **h–o** Immunoblotting, 3D-SIM, quantification, and FACS analyses of U2OS cells stably expressing the indicated constructs. Asterisk in (**h**), mGFP-containing degradation product. **i,j** Uncropped images used to generate Fig. 4d,e. Images in (**k**) show representative centrosomes for each group used for generating the quantified graphs in Fig. 4e (below). Note that "nebulous" mGFP-Vpr signals were manifest under MG132 treatment. Schematic diagrams (right) show the localization patterns of Cep152 and CP110 signals. Note the substantially increased fraction of cells with ≥3 Sas6 or ≥5 CP110 dot signals in cells expressing mGFP-Vpr, not mGFP-Vpr(1–79) in Fig. 4e. Unlike the previous finding [5], we did not observe a significant reduction in the signal intensity of CP110, a demonstrated target of the SKP1•CUL1•Cyclin F E3 ligase complex[6]. Also see the unchanged level of CP110 (Supplementary Fig. 3j). Vpr-induced centrosome amplification (marked by Cep152, whose localized signals tightly correlated with those of γ-tubulin signals) is cell cycle-independent (**m**). To monitor the cell cycle progression in cells expressing control vector, Vpr, or Vpr(1–79) (**m**), experiments were performed following the procedure provided in Source data. FACS data obtained from three independent experiments were analyzed by the FlowJo software 10.9.0. Bars in (**m**), mean of three experiments ± s.d. For the data in (**n**) and cell cycle analyses in (**o**), the same cells in (**m**) were treated with MG132 (3 h), thymidine (18 h), or nocodazole (18 h) following the schedule shown in (**n**). Asynchronously growing (Asyn) cells were left untreated. Cells with multiple centrosomes in (**n**) were quantified from three independent experiments. Per experiment, n ≥ 554 for mGFP/MG132 (total n = 1763); n ≥ 590 for mGFP-Vpr/MG132 (total n = 1894); n ≥ 609 for mGFP-Vpr(1–79)/MG132 (total n = 1921); n ≥ 534 for mGFP/Asyn (total n = 1688); n ≥ 549 for mGFP-Vpr/Asyn (total n = 1824); n ≥ 594 for mGFP-Vpr(1–79)/Asyn (total n = 1974); n ≥ 525 for mGFP/Thy (total n = 1608); n ≥ 524 for mGFP-Vpr/Thy (total n = 1990); n ≥ 501 for mGFP-Vpr(1–79)/Thy (total n = 1595); n ≥ 537 for mGFP/Noc (total n = 1684); n ≥ 525 for mGFP-Vpr/Noc (total n = 1675); n ≥ 517 for mGFP-Vpr(1–79)/Noc (total n = 1571). Bars in (**n**), mean of three experiments ± s.d.; P values, unpaired two-tailed *t*-tests; ns, not significant. Graphs in (**o**) were generated using FlowJo-analyzed data obtained from three independent experiments. Bars in (**o**), mean of three experiments ± s.d. All the FlowJo-analyzed data are provided in the Source data.

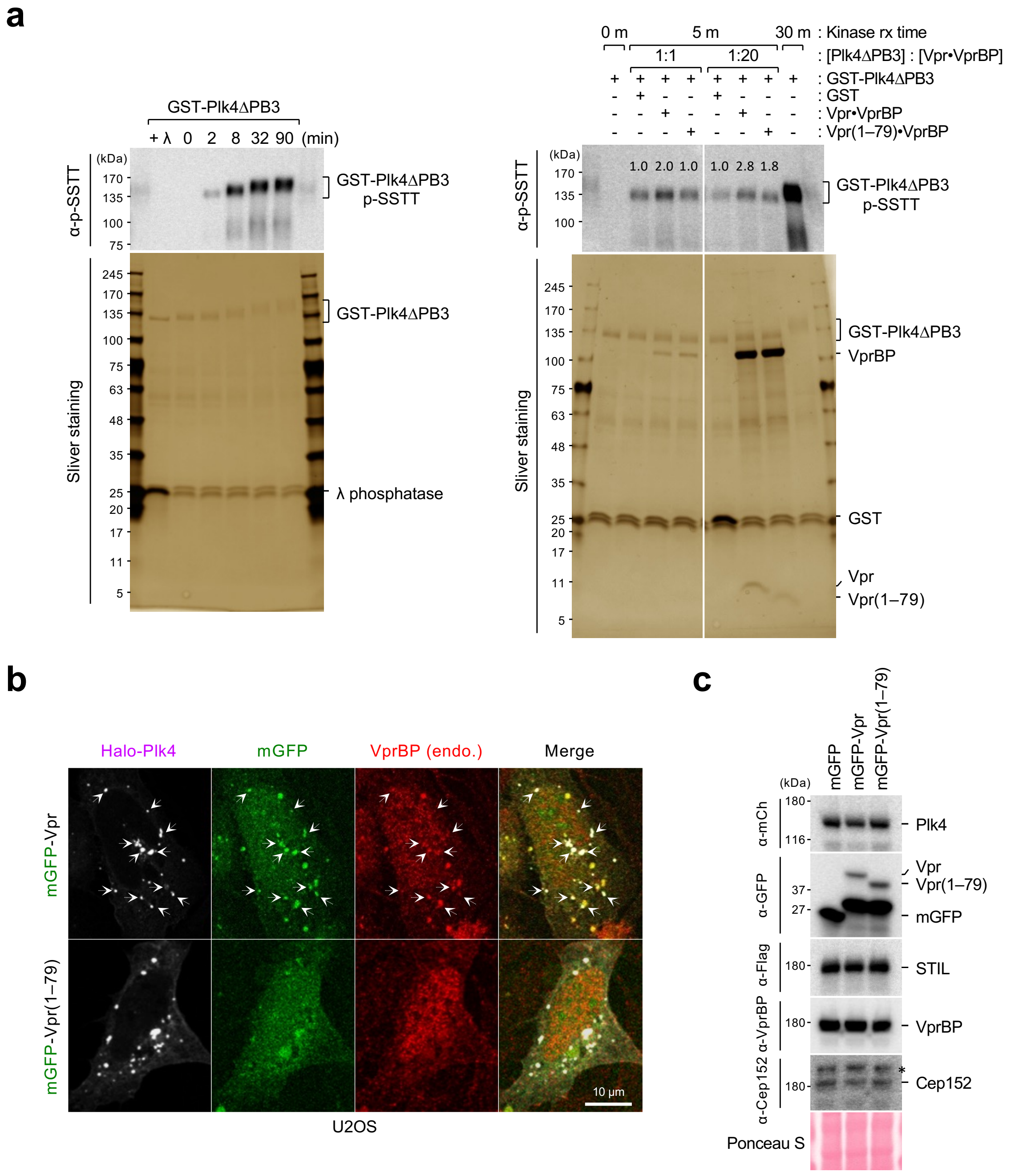

Supplementary Fig. 5. *In vitro* Plk4 kinase assays for determining the activational p-SSTT motif and *in vivo* Vpr•VprBP•Plk4 assemblies capable of generating the STIL p-S1108 epitope. a *In vitro* kinase assays and immunoblotting analyses performed with Plk4(1–836; ΔPB3) in the absence or presence of Vpr•VprBP or Vpr(1–79)•VprBP. Reactions were carried out to determine the *trans*-autophosphorylation activity of Plk4 (left) or the effect of the Vpr•VprBP complex on Plk4 kinase activity when provided at a 1:1 or 1:20 molar ratio (right). The degree of Plk4 activation was assessed by detecting the level of the p-STTT epitope generated in the Plk4 CPB[3,9]. Note that the provision of 20-fold excess Vpr•VprBP did not further increase Plk4 activity, suggesting that the activation step is already saturated. **b** Confocal images showing that mGFP-Vpr, but not mGFP-Vpr(1–79), generates cytosolic coassemblies with coexpressed Halo-Plk4 and endogenous VprBP in U2OS cells. White arrows indicate cytosolic assemblies containing colocalized Plk4, Vpr, and VprBP signals. **c** Immunoblotting of HEK293T cells

(CONTINUE)

coexpressing Plk4 and mGFP-Vpr or mGFP-Vpr(1–79) for 12 h. FLAG-STIL was also coexpressed to reliably quantify Plk4-depedent STIL phosphorylation at the S1108 motif[10]. Representative confocal images are shown in Fig. 5b. Quantified intensities determined from three independent experiments are provided in Fig. 5c. Note that like the results shown in (**b**), mGFP-Vpr(1–79) fails to efficiently generate cytosolic coassemblies with Plk4, although a low level of its signals colocalizing with Plk4 is apparent (see Fig. 5b,c). This is likely due to its ability to interact with VprBP, as shown in Fig. 3d, even without Plk4.

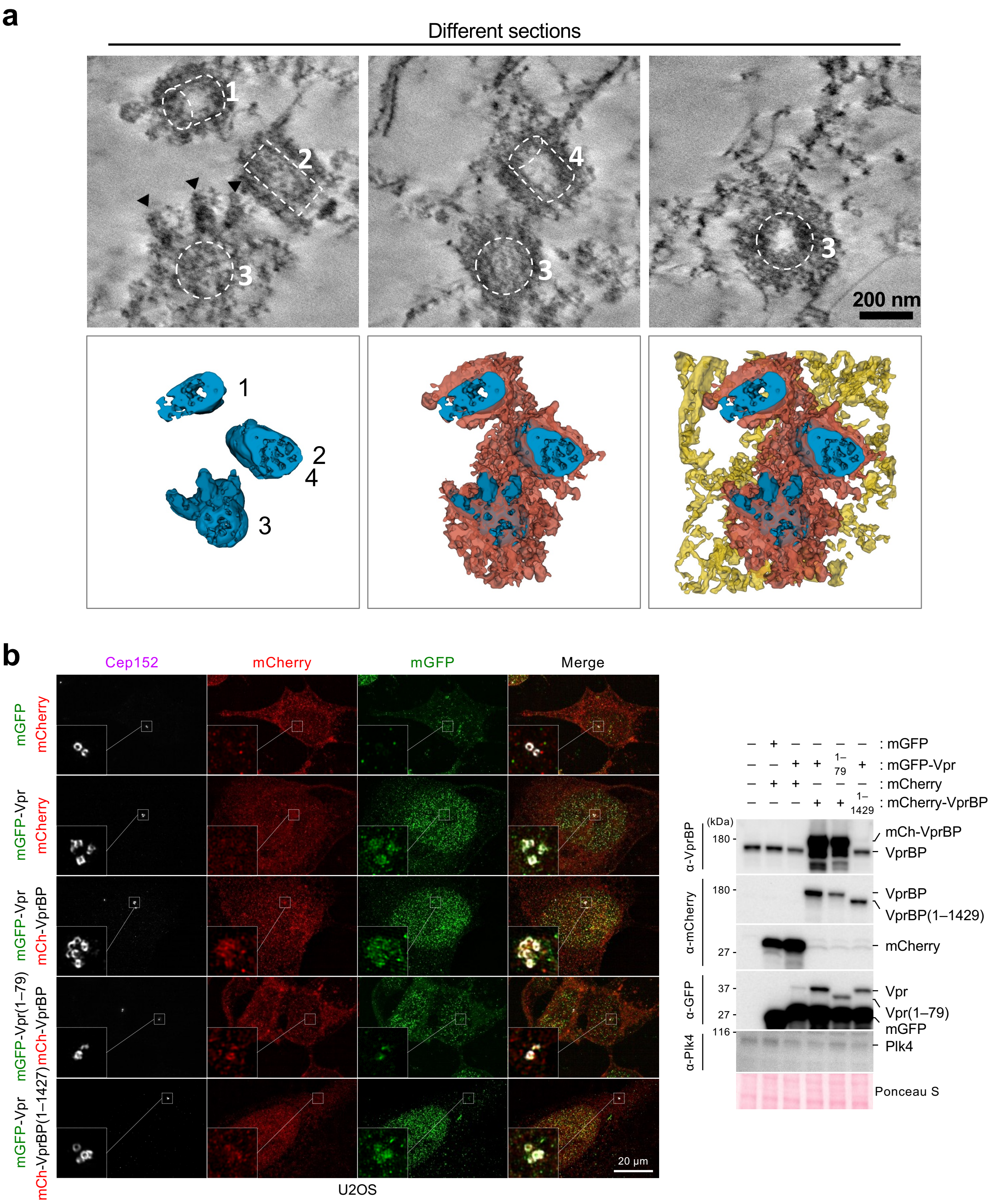
a
Different sections
1
2
3
4
200 nm
b
Cep152
mCherry
mGFP
Merge
mGFP
mCherry
mGFP-Vpr
mCherry
mGFP-Vpr
mCh-VprBP
mGFP-Vpr(1–79)
mCh-VprBP
mGFP-Vpr
mCh-VprBP(1–1427)
20 µm
U2OS
: mGFP
: mGFP-Vpr
: mCherry
: mCherry-VprBP
1–79
1–1429
(kDa)
α-VprBP
α-mCherry
α-GFP
α-Plk4
180
27
37
116
mCh-VprBP
VprBP
VprBP(1–1429)
mCherry
Vpr
Vpr(1–79)
mGFP
Plk4
Ponceau S

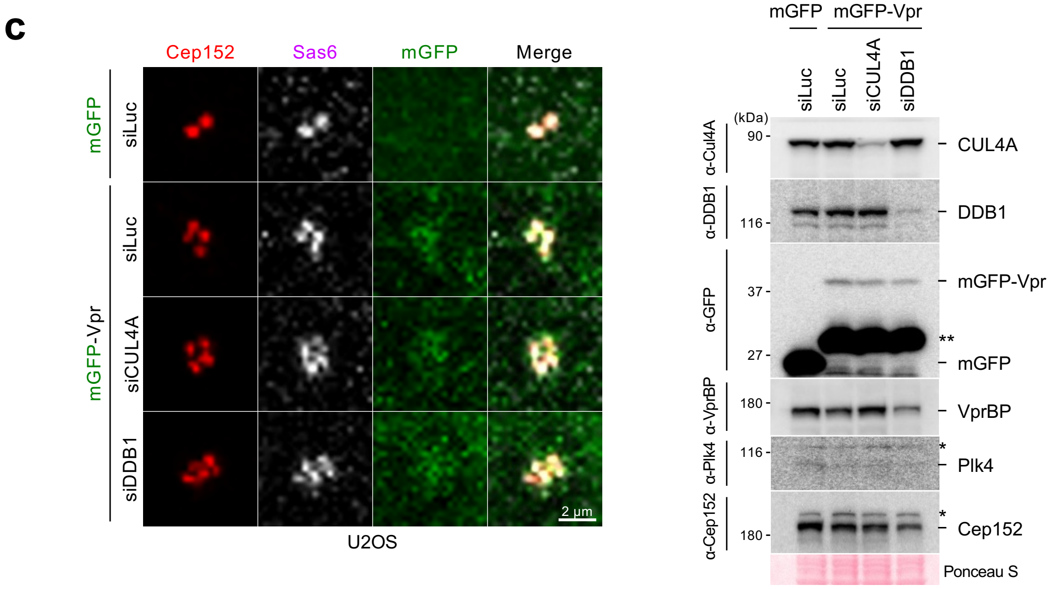

Supplementary Fig. 6. A TEM image showing Vpr-induced overduplicated centrioles and the requirement of Vpr CT and VprBP AR for this event. a Additional TEM tomography and segmentation of centrioles in a thymidine-treated (S phase) U2OS cell expressing mGFP-Vpr. Serial z-slices of the reconstructed tomogram (top row) show the presence of multiple disengaged centrioles (1 to 4) and the presence of distal appendages on centriole 3 (black arrowheads). Segmentation of centrioles (blue), their directly associated (red), and surrounding (yellow) cellular material are shown (bottom row). Centriole 4 is hidden because it is located behind centriole 2. **b** Confocal imaging and immunoblotting analyses for U2OS cells stably expressing the indicated constructs. Quantified data from these images are provided in Fig. 6b. Boxes, areas of enlargement; arrows, the relative positions of respective signals for easy comparison. **c** Confocal imaging and immunoblotting analyses for U2OS cells stably expressing mGFP-Vpr and silenced for luciferase or the indicated proteins by RNAi. Quantified data are provided in Fig. 6c. Asterisks, cross-reacting proteins; double asterisk, an mGFP-containing degradation product.

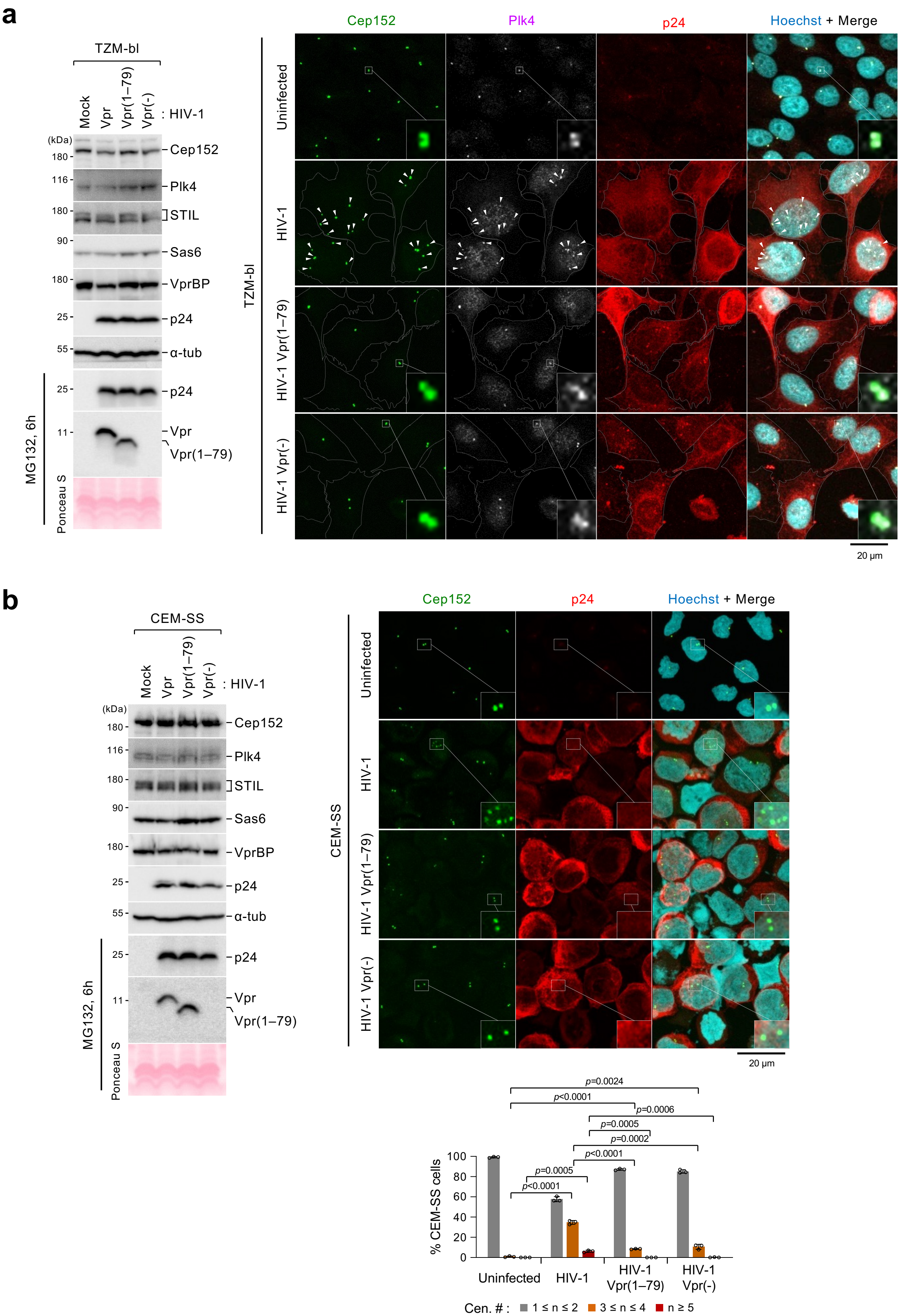
a
TZM-bl
Mock
Vpr
Vpr(1–79)
Vpr(-)
: HIV-1
(kDa)
180
116
180
90
180
25
55
25
11
Cep152
Plk4
STIL
Sas6
VprBP
p24
α-tub
p24
Vpr
Vpr(1–79)
MG132, 6h
Ponceau S
Cep152
Plk4
p24
Hoechst + Merge
TZM-bl
Uninfected
HIV-1
HIV-1 Vpr(1–79)
HIV-1 Vpr(-)
20 μm
b
CEM-SS
Mock
Vpr
Vpr(1–79)
Vpr(-)
: HIV-1
(kDa)
180
116
180
90
180
25
55
25
11
Cep152
Plk4
STIL
Sas6
VprBP
p24
α-tub
p24
Vpr
Vpr(1–79)
MG132, 6h
Ponceau S
Cep152
p24
Hoechst + Merge
CEM-SS
Uninfected
HIV-1
HIV-1 Vpr(1–79)
HIV-1 Vpr(-)
20 μm
p=0.0024
p<0.0001
p=0.0006
p=0.0005
p=0.0002
p<0.0001
p=0.0005
p<0.0001
% CEM-SS cells
100
80
60
40
20
0
Uninfected
HIV-1
HIV-1 Vpr(1–79)
HIV-1 Vpr(-)
Cen. # : 1 ≤ n ≤ 2 3 ≤ n ≤ 4 n ≥ 5

c

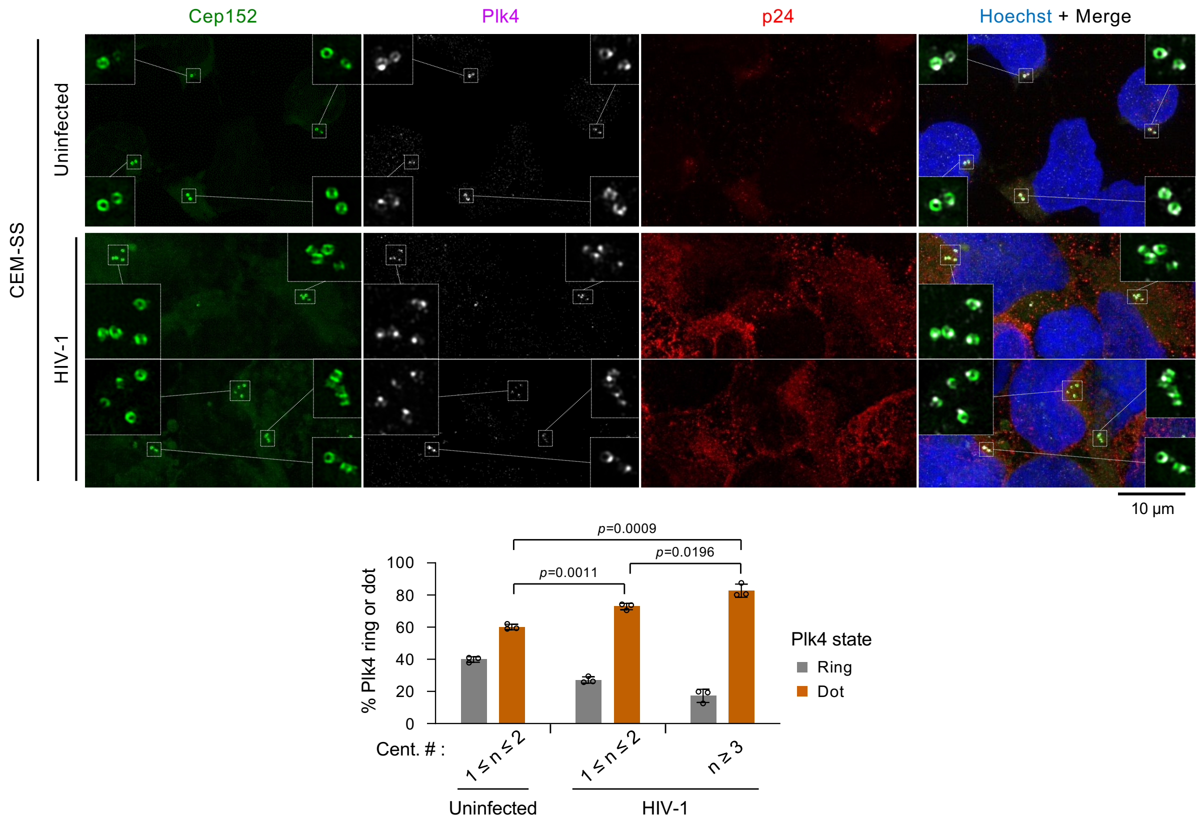

Cep152
Plk4
p24
Hoechst + Merge
CEM-SS
Uninfected
HIV-1
10 μm
p=0.0009
p=0.0011
p=0.0196
% Plk4 ring or dot
100
80
60
40
20
0
Cent. # :
1≤n≤2
1≤n≤2
n≥3
Uninfected
HIV-1
Plk4 state
Ring
Dot

d

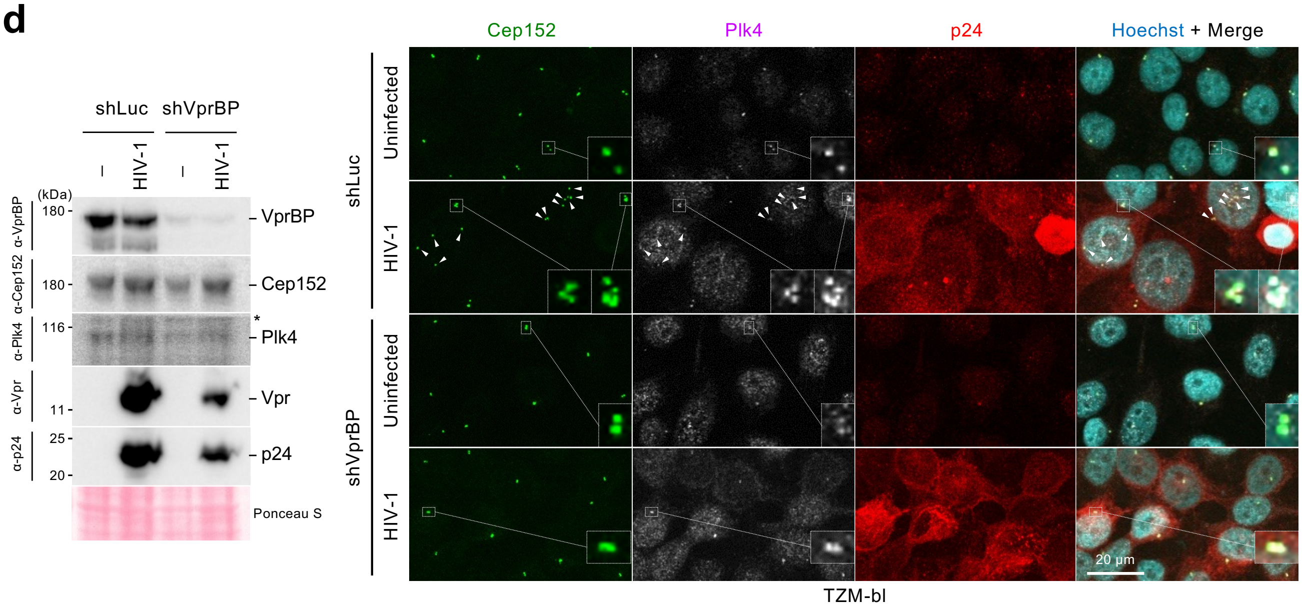

shLuc
shVprBP
–
HIV-1
–
HIV-1
(kDa)
α-VprBP
180
VprBP
α-Cep152
180
Cep152
α-Plk4
116
*
Plk4
α-Vpr
11
Vpr
α-p24
25
20
p24
Ponceau S
Cep152
Plk4
p24
Hoechst + Merge
shLuc
Uninfected
HIV-1
shVprBP
Uninfected
HIV-1
20 μm
TZM-bl

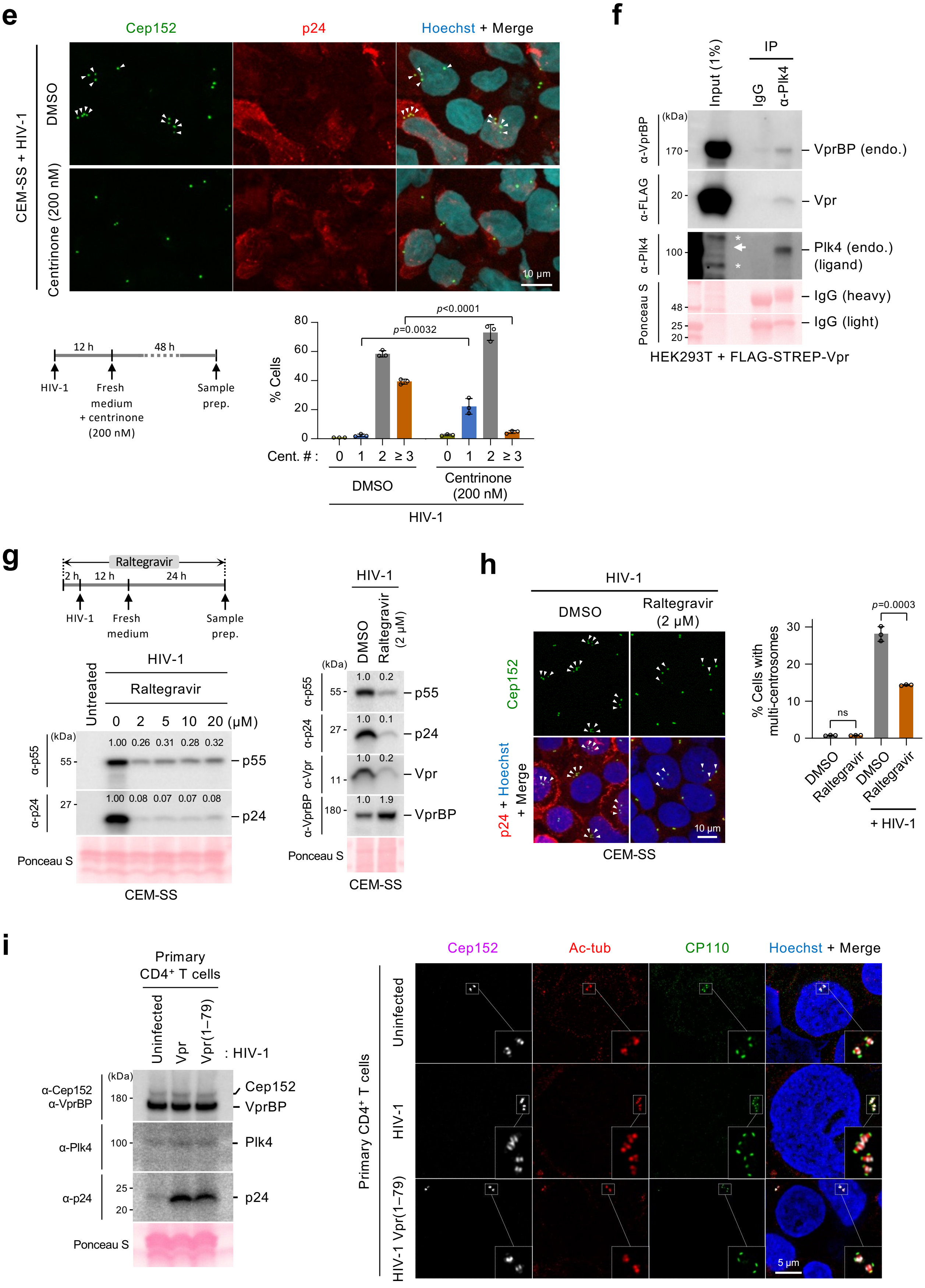
e
Cep152
p24
Hoechst + Merge
CEM-SS + HIV-1
DMSO
Centrinone (200 nM)
10 μm
12 h
48 h
HIV-1
Fresh medium + centrinone (200 nM)
Sample prep.
% Cells
p<0.0001
p=0.0032
Cent. # : 0 1 2 ≥ 3 0 1 2 ≥ 3
DMSO
Centrinone (200 nM)
HIV-1
f
IP
Input (1%)
IgG
α-Plk4
(kDa)
α-VprBP
α-FLAG
α-Plk4
Ponceau S
170
20
100
48
25
20
VprBP (endo.)
Vpr
Plk4 (endo.) (ligand)
IgG (heavy)
IgG (light)
HEK293T + FLAG-STREP-Vpr
g
Raltegravir
2 h
12 h
24 h
HIV-1
Fresh medium
Sample prep.
HIV-1
Untreated
Raltegravir
0 2 5 10 20 (μM)
(kDa)
α-p55
α-p24
55
27
1.00 0.26 0.31 0.28 0.32
1.00 0.08 0.07 0.07 0.08
p55
p24
Ponceau S
CEM-SS
HIV-1
DMSO
Raltegravir (2 μM)
(kDa)
α-p55
α-p24
α-Vpr
α-VprBP
55
27
11
180
1.0 0.2
1.0 0.1
1.0 0.2
1.0 1.9
p55
p24
Vpr
VprBP
Ponceau S
CEM-SS
h
HIV-1
DMSO
Raltegravir (2 μM)
Cep152
p24 + Hoechst + Merge
10 μm
CEM-SS
% Cells with multi-centrosomes
p=0.0003
ns
DMSO
Raltegravir
DMSO
Raltegravir
+ HIV-1
i
Primary CD4+ T cells
Uninfected
Vpr
Vpr(1–79)
: HIV-1
(kDa)
α-Cep152
α-VprBP
α-Plk4
α-p24
180
100
25
20
Cep152
VprBP
Plk4
p24
Ponceau S
Cep152
Ac-tub
CP110
Hoechst + Merge
Primary CD4+ T cells
Uninfected
HIV-1
HIV-1 Vpr(1–79)
5 μm

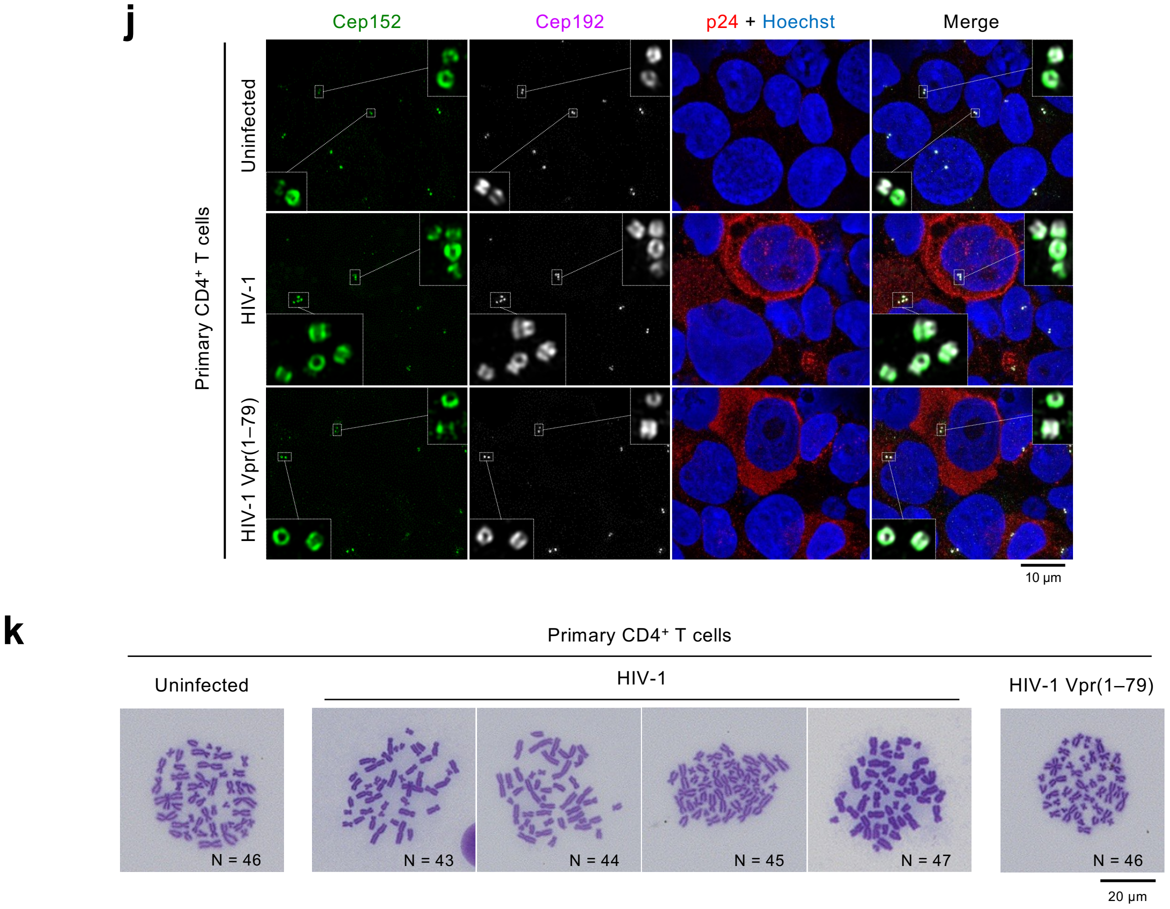

Supplementary Fig. 7. Vpr CT-dependent induction of centrosome amplification and aneuploidy in CD4$^+$ cells. a,b Immunoblotting and confocal imaging of TZM-bl (**a**) and CEM-SS (**b**) cells infected with the indicated viruses. To detect HIV-1-encoded Vpr and Vpr(1–79) by immunoblotting, the cells infected with the respective viruses were treated with MG132 for 6 hours before harvesting them to prepare total cell lysates. Arrowheads in (**a**), multiple Cep152 and Plk4 signals; boxes in (**a,b**), areas of enlargement. Centrosomes for cells in (**a,b**) were quantified among the p24$^+$ population by counting the Cep152 signals. The result from (**a**) is provided in Fig. 7a. Quantified data (graphs) were obtained from three independent experiments [per experiment, n ≥ 302 for uninfected cells (total n = 949); n ≥ 236 for HIV-1 (total n = 728); n ≥ 249 for HIV-1 Vpr(1–79) (total n = 767); n ≥ 223 for HIV-1 Vpr(–) (total n = 689)]. Bars, mean of three experiments ± s.d.; P values, unpaired two-tailed *t*-tests. Note that, like the TZM-bl cells in Fig. 7a, a small but significant fraction of CEM-SS cells infected with HIV-1 Vpr(1–79) or HIV-1 Vpr(-) still exhibit multiple centrosomes, suggesting that factor(s) other than Vpr could also contribute to the event causing centrosome overduplication. **c** Representative 3D-SIM images and quantified data for the CEM-SS cells left uninfected or infected with HIV-1 and immunostained with the indicated antibodies. Boxes, areas of enlargement. Quantification of HIV-1-infected cells was performed among the p24$^+$ population using images obtained from three independent experiments. Cells were grouped into two classes depending on the number of centrosomes as indicated. Per experiment, n ≥ 150 for uninfected cells (total n = 467); n ≥ 68 for HIV-1-infected cells with 1–2 centrosomes and n ≥ 93 for HIV-1-infected cells with ≥ 3 centrosomes (total n = 508). Bars, mean of three experiments ± s.d.; P values, unpaired two-tailed *t*-tests. **d** Immunoblotting and confocal imaging of TZM-bl cells infected with HIV-1 WT and silenced for control luciferase or VprBP. Asterisk, cross-reacting protein; boxes, areas of enlargement; arrowheads, multiple Cep152 and Plk4 signals. Quantified data are provided in Fig. 7b. **e** Confocal imaging and

(CONTINUE)
quantification of CEM-SS cells infected with HIV-1 for 12 h and then treated with fresh medium containing control DMSO or 200 nM centrinone for 48 h. Only the $p24^+$ population was quantified. Arrowheads, Cep152-marked centrosomes. Data are quantified from three independent experiments [per experiment, n ≥ 245 for control DMSO-treated cells (total n = 746); n ≥ 240 for centrinone-treated cells (total n = 742)]. Bars, mean of three experiments ± s.d.; P values, unpaired two-tailed *t*-tests. **f** Coimmunoprecipitation and immunoblotting analyses using HEK293T cells infected with lentiviruses expressing FLAG-STREP-Vpr. Endogenous Plk4 was immunoprecipitated with a mouse anti-Plk4 (6H5) antibody. Detection of coimmunoprecipitated proteins was performed with rabbit antibodies against the indicated proteins. The same membrane stained with Ponceau S is provided. Arrow, endogenous Plk4. **g,h** Immunoblotting, immunostaining, and quantification of cells with multiple centrosomes were carried out using HIV-1-infected CEM-SS cells treated with the indicated concentrations of raltegravir[11] for 38 h. HIV-1-encoded p24 and p55 levels remain unchanged between 2 μM and 20 μM of raltegravir treatment (**g**). Therefore, cells treated with 2 μM of raltegravir, which inhibits most of the integrase activity (judging from the diminished level of p24), were subjected to confocal analyses (**h**). CEM-SS cells with multiple centrosomes were quantified among the $p24^+$ population obtained from three independent experiments. Per experiment, n ≥ 1206 for control DMSO-treated cells (total n = 3822); n ≥ 1124 for raltegravir-treated cells (total n = 3699); n ≥ 511 for DMSO/HIV-1-infected cells (total n = 1615); n ≥ 512 for raltegravir/HIV-1-infected cells (total n = 1647). Arrowheads in (**h**, left), overduplicated centrosomes; Bars, mean of three experiments ± s.d.; P values, unpaired two-tailed *t*-tests; ns, not significant. **i** Immunoblotting and 3D-SIM imaging of primary $CD4^+$ T cells infected with the HIV-1 WT or Vpr(1–79) mutant for 8 h and cultured on the anti-CD3 and anti-CD28 antibody–coated plate for 4 days. Uncropped images used to generate Fig. 7d are provided. Boxes, areas of enlargement. **j** The same cells generated in (**i**) were immunostained and imaged. The Cep152 signals (centrosome marker) for each cell were quantified among the $p24^+$ population, and the results are provided in Fig. 7d (graph). Note that the level of the p24 signal is comparable between HIV-1 WT and the HIV-1 Vpr(1–79) mutant. **k** Representative chromosome spreads from the primary $CD4^+$ T cells infected with HIV-1 WT or its respective Vpr(1–79) mutant are shown. Quantified data are provided in Fig. 7e. Note that the preparation of chromosome spreads requires a dramatic swelling of mitotic cells, which increases cell volume up to five or more-fold[12,13]. Therefore, given that HIV-1-infected cells exhibit a few-fold increased cell volume, the efficiency of yielding well-spread mitotic chromosomes could be somewhat higher with HIV-1-infected cells than with uninfected cells.

Supplementary Table 1. Plasmid constructs used in this study

List	Protein	Expression vector	Restriction site	Source
Mammalian expression				
pKM4283	ZZ-TEV	pHR′.J-CMV-SV-puro (pKM2994)	SmaI/HindIII (e.f.), BamHI	This study
pKM4285	Plk4(581–884)	pHR′.J-CMV-SV-puro- ZZ-TEV (pKM4283)	SmaI/HindIII (e.f.), SalI/XhoI	This study
pKM4067	FLAG	pHR′.J-CMV-SV-puro (pKM2994)		Lab stock
pKM4591	Plk4(581–970)	pHR′.J-CMV-SV-puro-FLAG (pKM4067)	BamHI, SalI	[9]
pKM3445	Plk4	pCI-neo-FLAG (pKM2795)	PmeI, NotI	[14]
pKM3488	PLK4 (K41M)	pCI-neo-FLAG	PmeI, NotI	[15]
pKM3506	Plk4(1–581)	pCI-neo-FLAG	PmeI, NotI	[14]
pKM3507	Plk4(581–970)	pCI-neo-FLAG	PmeI, NotI	[14]
pKM3508	Plk4(581–884)	pCI-neo-FLAG	PmeI, NotI	[14]
pKM3509	Plk4(881–971)	pCI-neo-FLAG	PmeI, NotI	[14]
pKM3855	Plk4	pCI-neo-HA (pKM1209)	PmeI, NotI	[15]
pKM7582	Halo	pHR′.J-CMV-SV-puro (pKM2994)	EcoRI, AscI	This study
	Plk4		AscI, SalI	
pKM7089	Plk4	pHR′.J-CMV-SV-puro-mCherry (pKM6287)	BamHI, SalI	This study
pKM4358	VprBP	pCI-neo-HA	Pme I, XhoI	This study
pKM4548	VprBP	pCI-neo-GFP (pKM3828)	PmeI, NotI	This study
pKM4543	VprBP(1–1349)	pCI-neo-GFP	PmeI, NotI	This study
pKM4544	VprBP(1350–1507)	pCI-neo-GFP	PmeI, NotI	This study
pKM4545	VprBP(1401–1507)	pCI-neo-GFP	PmeI, NotI	This study
pKM4547	VprBP(1401–1470)	pCI-neo-GFP	PmeI, NotI	This study
pKM4546	VprBP(1401–1450)	pCI-neo-GFP	PmeI, NotI	This study
pKM5705	VprBP(1428–1488)	pCI-neo-GFP	PmeI, NotI	This study
pKM5684	VprBP(1428–1478)	pCI-neo-GFP	PmeI, NotI	This study
pKM5686	VprBP(1437–1478)	pCI-neo-GFP	PmeI, NotI	This study
pKM5721	VprBP(1446–1507)	pCI-neo-GFP	PmeI, NotI	This study
pKM5722	VprBP(1437–1507)	pCI-neo-GFP	PmeI, NotI	This study
pKM5723	VprBP(1428–1507)	pCI-neo-GFP	PmeI, NotI	This study
pKM5805	VprBP(1003–1507)	pCI-neo-GFP	PmeI, NotI	This study
pKM4617	VprBP(1003–1428)	pCI-neo-GFP	PmeI, NotI	This study
pKM4836	VprBP(1058–1507)	pCI-neo-GFP	PmeI, NotI	This study
pKM4630	VprBP	pCI-neo-FLAG	PmeI, XhoI	This study
pKM5284	VprBP(1–1428)	pCI-neo-FLAG	PmeI, NotI	This study
pKM5412	VprBP(1–1396)	pCI-neo-FLAG	PmeI, NotI	This study

pKM5283	VprBP(1–1349)	pCI-neo-FLAG	PmeI, NotI	This study
pKM7797	mCherry	pHR′.J-CMV-SV-puro (pKM2994)	EcoRI, BamHI	This study
pKM7574	mCherry-VprBP	pHR′.J-CMV-SV-puro	EcoRI, SalI	This study
pKM7794	mCherry-VprBP(1–1427)	pHR′.J-CMV-SV-puro	EcoRI, SalI	This study
pKM5330	Vpr (NL4-3 strain)*	pCI-neo-FLAG	PmeI, XhoI	This study
pKM5523	Vpr (NL4-3 strain)	pCI-neo-HA	PmeI, XhoI	This study
pKM5755	Vpr(K27M)	pCI-neo-HA	PmeI, XhoI	This study
pKM4753	Vpx (RCM-NG strain)	FLAG/HA vector		Monsef Benkirane (Institut de Génétique Humaine, Montpellier, France)
pKM4754	Vpx (ROD strain)	FLAG/HA vector		
pKM4755	Vpx (RCM-GAB strain)	FLAG/HA vector		
pKM4756	Vpx (MAC251 strain)	FLAG/HA vector		
pKM4757	Vpx (ROD strain)	FLAG vector		Michael Emerman (Fred Hutchinson Cancer Res. Center, WA, USA)
pKM4758	Vpr (Lai strain)	FLAG vector		
pKM4759	Vpr (NL4-3 strain)	pcDNA3.1-FLAG		Angela M. Gronenborn (University of Pittsburgh, PA, USA)
pKM8004	Vpr (89.6 strain)	pCI-neo-FLAG	PmeI, XhoI	This study
pKM8006	HIV-2 Vpr (NWK08 strain)	pCI-neo-FLAG	PmeI, XhoI	This study
pKM7799	mGFP-STREP-TEV	pHR′.J-CMV-SV-puro	EcoRI, BamHI	This study
pKM7653	Vpr (NL4-3 strain)	pHR′.J-CMV-SV-puro-mGFP-STREP-TEV (pKM7652)	AscI, BamHI	This study
pKM7654	Vpr(1–63)	pHR′.J-CMV-SV-puro-mGFP-STREP-TEV	AscI, BamHI	This study
pKM7655	Vpr(1–82)	pHR′.J-CMV-SV-puro-mGFP-STREP-TEV	AscI, BamHI	This study
pKM7666	Vpr(51–96)	pHR′.J-CMV-SV-puro-mGFP-STREP-TEV	AscI, BamHI	This study
pKM7667	Vpr(75–96)	pHR′.J-CMV-SV-puro-mGFP-STREP-TEV	AscI, BamHI	This study
pKM7798	mGFP	pHR′.J-CMV-SV-puro	EcoRI, BamHI	This study

pKM7618	Vpr (NL4-3 strain)	pHR′.J-CMV-SV-puro-mGFP (pKM7410)	AscI, BamHI	This study
pKM7792	Vpr(1–79)	pHR′.J-CMV-SV-puro-mGFP	AscI, BamHI	This study
pKM8001	Vpr (89.6 strain)	pHR′.J-CMV-SV-puro-mGFP	AscI, BamHI	This study
pKM8003	HIV-2 Vpr (NWK08 strain)	pHR′.J-CMV-SV-puro-mGFP	AscI, BamHI	This study
pKM7632	Vpr (NL4-3 strain)	pHR′.J-CMV-SV-puro-FLAG-STREP-TEV (pKM7631)	AscI, BamHI	This study
pKM4354	DDB1	pCI-neo-HA	PmeI, NotI	This study
pKM3676	Plk1	pCI-neo-FLAG	PmeI, NotI	This study
pKM2805	Cep78	pCI-neo-FLAG	PmeI, NotI	This study
pKM1226	Cep78	pCI-neo-HA	EcoRV/SmaI, NotI	This study
pKM7084	STIL	pHR′.J-CMV-SV-puro-FLAG	AscI, PmeI	This study
pKM7743	shLuc	pLKO.1 hygro (Addgene #24150)	AgeI, EcoRI	This study
pKM7744	shPlk4	pLKO.1 hygro	AgeI, EcoRI	This study
pKM7746	shVprBP	pLKO.1 hygro	AgeI, EcoRI	This study
pKM7595	Vpr (NL4-3 strain)	pNLenv1		This study
pKM8247	Vpr(1–79)	pNLenv1	A stop codon after S79	This study
pKM7628	Vpr(-)	pNLenv1	A stop codon after E2	This study
pKM6453	pCMV-VSVG			Cell Biolabs (Cat # RV-110)
E. coli and Sf9 expression				
pKM6934	VprBP(1446–1507)	pETDuet1-His-MBP-TEV	PmeI, NotI	This study
	Plk4(581–808)		NdeI, XhoI	
pKM5440	Plk4(581–808)	pET28b(+)-His6	BamHI, SalI	This study
pKM3677	Plk4(581–808)	His6-MBP-TEV	NdeI, XhoI	[15]
pKM7608	Plk4(468–970)	His6-MBP-TEV	NdeI, XhoI	This study
pKM7643	Plk4(468–970)	His6-MBP-TEV-D5-FLAG	NdeI, XhoI	This study
pKM7671	Plk4(581–970)	His6-MBP-TEV-D5-FLAG	NdeI, XhoI	This study
pKM7672	Plk4(468–807)	His6-MBP-TEV-D5-FLAG	NdeI, XhoI	This study
pKM4601	Plk4	His6-MBP-TEV	NdeI, XhoI	This study
pKM4602	Plk4(K41M)	His6-MBP-TEV	NdeI, XhoI	This study
pKM7669	VprBP(1057–1507)-STREP-P10-Vpr	pTriEx-4-his-MBP-TEV	AscI, XhoI	This study
pKM7731	VprBP(1021–1507)-STREP-P10-Vpr	pTriEx-4-his-MBP-TEV	AscI, XhoI	This study

pKM7860	VprBP(1021–1507)-STREP-P10-Vpr(1–79)	pTriEx-4-his-MBP-TEV	AscI, XhoI	This study
pKM6931	Vpr(H78C)-His	NusA-TEV (pET43a)	EcoRI, HindIII	[8]

*The Vpr construct (M5330) was used to generate all the Vpr-related constructs listed in this table. The ORF of this construct is identical to the infectious molecular clone pNL4-3[16], M4521 (pcDNA3.1-FLAG-Vpr; a gift of J. I. Park, MD Anderson Cancer Center, TX), M4759 (pcDNA3.1-FLAG-Vpr; a gift of A. M. Gronenborn, University of Pittsburgh, PA), and M5286 (pCEP4-HA-Vpr; a gift of J. Luban, University of Massachusetts Medical School, MA).

Supplementary Table 2. siRNA sequences used in this study

Target Gene	Sequence (nt positions from the start codon)	Type	Source
Luciferase	CGTACGCGGAATACTTCGA	Synthetic Lentivirus	[17]
VprBP	CGAGAAACTGAGTCAAATGAA	Synthetic Lentivirus	[18]
Plk4	CTCCTTTCAGACATATAAG	Synthetic Lentivirus	[7]
DDB1	siRNA Oligo Duplex (Locus ID 1642)	Synthetic	Origene (SR301160)[19]
CUL4A	ON-TARGETplus siRNA-SMARTpool	Synthetic	Dharmcon (L-012610)[19]
βTrCP	GTGGAATTTGTGGAACATCTT	Synthetic	[20]
Cep78	GGCTGAGAGTCTTCGCTAT<br>GGTCGTTCTGGATATAAGA	Synthetic	[21]

Supplementary Table 3. Antibodies used in this study

Antibodies	Species	Source	Cat #	Exp. dilution
Primary antibodies				
Anti-HA (3F10)	Rat	Roche	11867431001	1:1000 (IB), 2 μg (IP)
Anti-HA-HRP conjugate	Mouse	Cell Signaling	#2999	1:3000 (IB)
Anti-FLAG (M2)	Mouse	Millipore Sigma	F1804	1:1000 (IB), 1:200 (IF)
Anti-FLAG M2 Affinity Gel	Mouse	Millipore Sigma	A2220	
Anti-FLAG (M2)-HRP conjugate	Mouse	Millipore Sigma	A8592	1:10000 (IB)
Anti-GFP	Rabbit	Santa Cruz	sc-8334	1:1000 (IB)
Anti-GFP (B-2)	Mouse	Santa Cruz	sc-9996	1:1000 (IB), 1:400 (IF)
Anti-GFP (B-2)-HRP conjugate	Mouse	Santa Cruz	sc-9996HRP	1:3000 (IB)
Anti-GFP	Rat	MBL international	D153-3	2 μg (IP)
Anti-mCherry	Rabbit	Proteintech	26765-1-AP	1:1000 (IB)

Alexa Fluor 594-conjugated mCherry	Rat	Thermo Fisher Scientific	M11240	1:500 (IF)
Anti-Cdc25C (C-20)	Rabbit	Santa Cruz	sc-327	1:1000 (IB)
Anti-VprBP	Rabbit	Bethyl Laboratories	A301-887A	1:150 (IF)
Anti-VprBP	Rabbit	Millipore Sigma	ABS1583	1:1000 (IB), 1:150 (IF)
Anti-UNG2 (OTI2C12)	Mouse	Origene	TA503563S	1:1000 (IB)
Anti-CP110	Rabbit	Bethyl	A301-343A	1:1000 (IB)
Anti-CP110	Rabbit	Proteintech	12780-1-AP	1:2000 (IF)
Anti-Plk4 (6H5)	Mouse	Millipore Sigma	MABC544	1:1000 (IB) 1:200 (IF)
Anti-Plk4 (581–970)	Rabbit	[14]	Lab supply	1:200 (IF)
Anti-Plk4 p-SSTT	Rabbit		Lab supply	1:1000 (IB)
Anti-βTrCP (C-6)	Mouse	Santa Cruz	sc-390629	1:1000 (IB)
Anti-Cep152 (GT1285)	Mouse	GeneTex	GTX631486	1:400 (IF)
Anti-Cep152 (491–810)	Rabbit	[14]	Lab supply	1:1000 (IB) 1:300 (IF)
Anti-Cep152 (1350–1654)	Rabbit	[14]	Lab supply	1:1000 (IB)
Alexa Fluor 647-conjugated Cep152 (491-810)	Rabbit	[14]	Lab supply	1:200 (IF)
Alexa Fluor 488-conjugated Cep152 (491-810)	Rabbit	[14]	Lab supply	1:200 (IF)
Anti-Cep192 (1-647)	Rabbit	[14]	Lab supply	1:200 (IF)
Anti-acetylated tubulin (6-11B-1)	Mouse	Millipore Sigma	T7451	1:200 (IF)
Anti-Sas6 (404-657)	Mouse	Santa Cruz	sc-81431	1:200 (IF)
Anti-CUL4A(EPR3198)	Rabbit	Abcam	ab92554	1:1000 (IB)
Anti-DDB1	Rabbit	Cell Signaling	#5428	1:1000 (IB)
Anti-α-tubulin (B-5-1-2)	Mouse	Sigma	T6074	1:1000 (IB) 1:200 (IF)
Anti-γ-tubulin (c-20)	Goat	Santa Cruz	sc-7396	1:200 (IF)
Anti-γ-tubulin (GTU-88)	Mouse	Millipore-Sigma	T-5326	1:200 (IF)
Anti-STIL	Rabbit	Abcam	ab89314	1:1000 (IB)
Anti-STIL p-S1108	Rabbit		Lab supply	1:400 (IF)
Anti-Cep78	Rabbit	Bethyl Laboratories	A301-800A	1:500 (IB)
Anti-p24 (#24-3)	Mouse	NIH HIV Reagent Program	ARP-6458	1:1000 (IB)
HIV-1 IG (pooled human serum from HIV-1^+ individuals)	Human	NIH HIV Reagent Program	ARP-3957	1:10000 (IB)
Anti-Vpr (8D1)	Mouse	Cosmo Bio	CAC-NCG-M01-1	1:1000 (IB)

Anti-Vpr cpz	Rabbit	Unpublished	Home made	1:2500 (IB)
Anti-Cep63	Rabbit	Millipore Sigma	06-1292	1:1000 (IB)<br>1:200 (IF)
Anti-Cep192 (1–647)	Rabbit	[14]	Lab supply	1:200 (IF)
Anti-CD3	Mouse	Invitrogen	16-0037-81	2 µg/mL (coating)
Anti-CD28	Mouse	Invitrogen	14-0289-82	2 µg/mL (coating)
Secondary antibodies (Immunofluorescence)				
Anti-rabbit IgG Alexa Fluor 488-conjugated	Donkey	Invitrogen	A-21206	1:300 (IF)
Anti-mouse IgG Alexa Fluor 594-conjugated	Donkey	Invitrogen	A-21203	1:300 (IF)
Anti-rabbit IgG Alexa Fluor 594-conjugated	Donkey	Invitrogen	A-21207	1:300 (IF)
Anti-mouse IgG Alexa Fluor 647-conjugated	Donkey	Invitrogen	A-31571	1:300 (IF)
Anti-rabbit IgG Alexa Fluor 647-conjugated	Donkey	Invitrogen	A-31573	1:300 (IF)
F(ab')2 Anti-mouse IgG Alexa Fluor 488-conjugated	Goat	Invitrogen	A-11017	1:400 (IF)
F(ab')2 Anti-mouse IgG Alexa Fluor 594-conjugated	Goat	Invitrogen	A-11020	1:400 (IF)
F(ab')2 Anti-rabbit IgG Alexa Fluor 488-conjugated	Goat	Invitrogen	A-11070	1:400 (IF)
F(ab')2 Anti-rabbit IgG Alexa Fluor 594-conjugated	Goat	Invitrogen	A-11072	1:400 (IF)
F(ab')2 Anti-goat IgG Alexa Fluor 488-conjugated	Rabbit	Invitrogen	A-21222	1:400 (IF)
Secondary antibodies (Immunoblot)				
Anti-mouse IgG HRP-conjugated	Donkey	GE Healthcare	NA9310	1:3000 (IB)
Anti-rabbit IgG HRP-conjugated	Donkey	GE Healthcare	NA9340	1:3000 (IB)
Anti-rat IgG HRP-conjugated	Goat	Thermo Fisher Scientific	#31470	1:10000 (IB)
Anti-human IgG HRP-conjugated				1:10000 (IB)

Supplementary Information References

1 Hleyhel, M. *et al.* Risk of AIDS-defining cancers among HIV-1-infected patients in France between 1992 and 2009: results from the FHDH-ANRS CO4 cohort. *Clin Infect Dis* **57**, 1638-1647 (2013). https://doi.org:10.1093/cid/cit497

2 Agarwal, N. *et al.* HIV-1 Vpr induces adipose dysfunction in vivo through reciprocal effects on PPAR/GR co-regulation. *Sci Transl Med* **5**, 213ra164 (2013). https://doi.org:10.1126/scitranslmed.3007148

3 Park, J. E. *et al.* Autophosphorylation-induced self-assembly and STIL-dependent reinforcement underlie Plk4's ring-to-dot localization conversion around a human centriole. *Cell Cycle* **19**, 3419-3436 (2020).

4 Ahn, J., Vu, T., Novince, Z., Guerrero-Santoro, J., Rapic-Otrin, V. & Gronenborn, A. M. HIV-1 Vpr loads uracil DNA glycosylase-2 onto DCAF1, a substrate recognition subunit of a cullin 4A-ring E3 ubiquitin ligase for proteasome-dependent degradation. *J Biol Chem* **285**, 37333-37341 (2010). https://doi.org:10.1074/jbc.M110.133181

5 Hossain, D., Javadi Esfehani, Y., Das, A. & Tsang, W. Y. Cep78 controls centrosome homeostasis by inhibiting EDD-DYRK2-DDB1(Vpr)(BP). *EMBO Rep* **18**, 632-644 (2017). https://doi.org:10.15252/embr.201642377

6 D'Angiolella, V. *et al.* SCF(Cyclin F) controls centrosome homeostasis and mitotic fidelity through CP110 degradation. *Nature* **466**, 138-142 (2010). https://doi.org:10.1038/nature09140

7 Guderian, G., Westendorf, J., Uldschmid, A. & Nigg, E. A. Plk4 trans-autophosphorylation regulates centriole number by controlling betaTrCP-mediated degradation. *J Cell Sci* **123**, 2163-2169 (2010). https://doi.org:10.1242/jcs.068502

8 Wu, Y. *et al.* The DDB1-DCAF1-Vpr-UNG2 crystal structure reveals how HIV-1 Vpr steers human UNG2 toward destruction. *Nat Struct Mol Biol* **23**, 933-940 (2016). https://doi.org:10.1038/nsmb.3284

9 Park, J. E., Zhang, L., Bang, J. K., Andresson, T., DiMaio, F. & Lee, K. S. Phase separation of Polo-like kinase 4 by autoactivation and clustering drives centriole biogenesis. *Nat Commun* **10**, 4959 (2019). https://doi.org:10.1038/s41467-019-12619-2

10 Ohta, M. *et al.* Direct interaction of Plk4 with STIL ensures formation of a single procentriole per parental centriole. *Nat Commun* **5**, 5267 (2014). https://doi.org:10.1038/ncomms6267

11 Anker, M. & Corales, R. B. Raltegravir (MK-0518): a novel integrase inhibitor for the treatment of HIV infection. *Expert Opin Investig Drugs* **17**, 97-103 (2008). https://doi.org:10.1517/13543784.17.1.97

12 Ami, D. *et al.* Role of water in chromosome spreading and swelling induced by acetic acid treatment: a FTIR spectroscopy study. *Eur J Histochem* **58**, 2330 (2014). https://doi.org:10.4081/ejh.2014.2330

13 Claussen, U. *et al.* Demystifying chromosome preparation and the implications for the concept of chromosome condensation during mitosis. *Cytogenet Genome Res* **98**, 136-146 (2002). https://doi.org:10.1159/000069817

14 Kim, T.-S. *et al.* Hierarchical recruitment of Plk4 and regulation of centriole biogenesis by two centrosomal scaffolds, Cep192 and Cep152. *Proc Natl Acad Sci USA* **110**, E4849-4857 (2013).

15 Park, S. Y. *et al.* Molecular basis for unidirectional scaffold switching of human Plk4 in centriole biogenesis. *Nat Struct Mol Biol* **21**, 696-703 (2014). https://doi.org:10.1038/nsmb.2846
16 Adachi, A. *et al.* Production of acquired immunodeficiency syndrome-associated retrovirus in human and nonhuman cells transfected with an infectious molecular clone. *J Virol* **59**, 284-291 (1986). https://doi.org:10.1128/JVI.59.2.284-291.1986
17 Elbashir, S. M., Harborth, J., Lendeckel, W., Yalcin, A., Weber, K. & Tuschl, T. Duplexes of 21-nucleotide RNAs mediate RNA interference in cultured mammalian cells. *Nature* **411**, 494-498 (2001).
18 Kyei, G. B., Cheng, X., Ramani, R. & Ratner, L. Cyclin L2 is a critical HIV dependency factor in macrophages that controls SAMHD1 abundance. *Cell Host Microbe* **17**, 98-106 (2015). https://doi.org:10.1016/j.chom.2014.11.009
19 Jang, S. M. *et al.* The RepID-CRL4 ubiquitin ligase complex regulates metaphase to anaphase transition via BUB3 degradation. *Nat Commun* **11**, 24 (2020). https://doi.org:10.1038/s41467-019-13808-9
20 Guardavaccaro, D. *et al.* Control of meiotic and mitotic progression by the F box protein beta-Trcp1 in vivo. *Dev Cell* **4**, 799-812 (2003).
21 Brunk, K. *et al.* Cep78 is a new centriolar protein involved in Plk4-induced centriole overduplication. *J Cell Sci* **129**, 2713-2718 (2016). https://doi.org:10.1242/jcs.184093
