# Supplementary material for: Centrosome amplification and aneuploidy driven by the HIV-1-induced Vpr•VprBP•Plk4 complex in CD4+ T cells
Source: Nat Commun. 2024 Mar 5;15:2017. doi: 10.1038/s41467-024-46306-8 (PMC10914751; doi:10.1038/s41467-024-46306-8)
Supplement: Supplementary file 3 — Reporting Summary [file 41467_2024_46306_MOESM3_ESM.pdf]

nature portfolio

Corresponding author(s): Kyung S. Lee

Last updated by author(s): Feb 4, 2024

Reporting Summary

Nature Portfolio wishes to improve the reproducibility of the work that we publish. This form provides structure for consistency and transparency in reporting. For further information on Nature Portfolio policies, see our Editorial Policies and the Editorial Policy Checklist.

Statistics

For all statistical analyses, confirm that the following items are present in the figure legend, table legend, main text, or Methods section.

n/a	Confirmed	
☐	☒	The exact sample size (*n*) for each experimental group/condition, given as a discrete number and unit of measurement
☐	☒	A statement on whether measurements were taken from distinct samples or whether the same sample was measured repeatedly
☐	☒	The statistical test(s) used AND whether they are one- or two-sided<br>*Only common tests should be described solely by name; describe more complex techniques in the Methods section.*
☒	☐	A description of all covariates tested
☒	☐	A description of any assumptions or corrections, such as tests of normality and adjustment for multiple comparisons
☐	☒	A full description of the statistical parameters including central tendency (e.g. means) or other basic estimates (e.g. regression coefficient) AND variation (e.g. standard deviation) or associated estimates of uncertainty (e.g. confidence intervals)
☐	☒	For null hypothesis testing, the test statistic (e.g. *F*, *t*, *r*) with confidence intervals, effect sizes, degrees of freedom and *P* value noted<br>*Give P values as exact values whenever suitable.*
☒	☐	For Bayesian analysis, information on the choice of priors and Markov chain Monte Carlo settings
☒	☐	For hierarchical and complex designs, identification of the appropriate level for tests and full reporting of outcomes
☒	☐	Estimates of effect sizes (e.g. Cohen's *d*, Pearson's *r*), indicating how they were calculated

Our web collection on statistics for biologists contains articles on many of the points above.

Software and code

Policy information about availability of computer code

Data collection	Zeiss ELYRA S1 super-resolution microscope (Zeiss), 120 kV Talos L120C TEM (Thermo Fisher Scientific), Ceta 16M CCD camera, Model 2020 advanced tomography holder (Fischione), Keyence inverted fluorescence phase contrast microscope BZX710, chemiluminescence imager (ChemiDoc™ Imaging Systems, Bio-Rad Laboratories), Cytiva AKTA pure chromatography system (Cytiva), Refeyn TwoMP mass photometer (Refeyn Ltd.), BD LSRFortessa flow cytometer, LTQ linear ion trap mass spectrometer (Thermo Electron) for MS/MS and MS/MS/MS analysis (nanoLC-MS2-MS3)
Data analysis	Zeiss Zen v2.1 software (Zeiss), GraphPad Prism8, SerialEM 82 software, Etomo in the IMOD software package, version 4.11, Etomo, Quicktime, 3D Slicer Version 5.0.2, ImageJ Fiji (NIH), Image Lab (Bio-Rad), Cytiva Unicorn 7.6 (Cytiva), DiscoverMP program (Refeyn Ltd.), Flowjo (BD Biosciences), FlowJo software, Sequest (Thermo Electron) for MS2 and MS3 data search.

For manuscripts utilizing custom algorithms or software that are central to the research but not yet described in published literature, software must be made available to editors and reviewers. We strongly encourage code deposition in a community repository (e.g. GitHub). See the Nature Portfolio guidelines for submitting code & software for further information.

Data

Policy information about availability of data

All manuscripts must include a data availability statement. This statement should provide the following information, where applicable:

- Accession codes, unique identifiers, or web links for publicly available datasets
- A description of any restrictions on data availability
- For clinical datasets or third party data, please ensure that the statement adheres to our policy

All the raw data used for quantification and statistical analyses are provided as Source Data file and mass spectrometry data are deposited in ProteomeXchange (PRIDE). Accession number (MSV000094032) is provided.

Research involving human participants, their data, or biological material

Policy information about studies with human participants or human data. See also policy information about sex, gender (identity/presentation), and sexual orientation and race, ethnicity and racism.

Reporting on sex and gender	Study participants were randomly selected without considering sex and gender
Reporting on race, ethnicity, or other socially relevant groupings	Study participants were randomly selected without considering ethnicity.
Population characteristics	N/A
Recruitment	Study participants were recruited in a random fashion
Ethics oversight	N/A

Note that full information on the approval of the study protocol must also be provided in the manuscript.

Field-specific reporting

Please select the one below that is the best fit for your research. If you are not sure, read the appropriate sections before making your selection.

☒ Life sciences ☐ Behavioural & social sciences ☐ Ecological, evolutionary & environmental sciences

For a reference copy of the document with all sections, see nature.com/documents/nr-reporting-summary-flat.pdf

Life sciences study design

All studies must disclose on these points even when the disclosure is negative.

Sample size	The sample size was determined based on the size of the standard deviation and the reproducibility of the data. All the sample numbers are provided. Statistical analyses were performed with data obtained from at least three independent experiments. For samples from people living with HIV-1, a total of only 14 samples (four of them were paired samples with and without ART) were analyzed because of difficulty of finding these clinical samples. The sex and/or gender, which was determined based on self-report, was not considered in the study design. Age, one of the important factors for HIV-1 pathogenesis, was considered. A graph showing the age distribution of study participants is provided in Supplementary Fig. 1f. The study was performed in accordance with NIH clinical protocols (NIH IRB FWA00005897). All study participants provided written informed consent for the research.
Data exclusions	No data points were excluded from quantifications.
Replication	Except the results obtained from human subjects with HIV-1, all experiments were performed at least three times and results were successfully reproduced. In all cases, the results reliably support conclusions stated in the manuscript.
Randomization	For quantifying immunostained samples and chromosome spreads, images were taken randomly from undefined areas. All the samples acquired were quantified to generate the data shown in the manuscript. Human samples from healthy people and people living with HIV-1 were randomly chosen as long as they belong to a similar age group. Sex and/or gender were not considered in the study design.
Blinding	Acquisition of images and quantifications were conducted in a blinded manner.

Reporting for specific materials, systems and methods

We require information from authors about some types of materials, experimental systems and methods used in many studies. Here, indicate whether each material, system or method listed is relevant to your study. If you are not sure if a list item applies to your research, read the appropriate section before selecting a response.

Materials & experimental systems

n/a	Involved in the study
☐	☒ Antibodies
☐	☒ Eukaryotic cell lines
☒	☐ Palaeontology and archaeology
☒	☐ Animals and other organisms
☐	☒ Clinical data
☒	☐ Dual use research of concern
☒	☐ Plants

Methods

n/a	Involved in the study
☒	☐ ChIP-seq
☐	☒ Flow cytometry
☒	☐ MRI-based neuroimaging

Antibodies

Antibodies used

All the antibodies used in this study are listed in the Supplementary Table 3 with source information, catalog numbers, and dilution factors. All antibodies are available upon request.

Validation

All the antibodies used in this study were either internally controlled, validated by siRNA, or previously published. For the VprBP, antibody, an antibody blocking was performed to confirm the validity of the antibody. No antibody was used without validation.

Eukaryotic cell lines

Policy information about cell lines and Sex and Gender in Research

Cell line source(s)

U2OS and HEK293T cells were purchased from American Type Culture Collection (ATCC) and cultured as recommended by ATCC. CEM-SS and TZM-bl cells were obtained through the NIH HIV Reagent Program. human CD4+ T cells were purified and validated by flow cytometry. PBMCs from HIV-positive patients were obtained through the NIH clinical center (approved by the Institutional Review Board of NIH; NIH/NIAID IRB FWA00005897).

Authentication

Cells have been authenticated by the vendor and used as low passage cell lines for experiments. No further authentication was performed for cell lines from ATCC.

Mycoplasma contamination

No mycoplasma contamination was found.

Commonly misidentified lines
(See ICLAC register)

No commonly misidentified cell lines were used.

Clinical data

Policy information about clinical studies

All manuscripts should comply with the ICMJEguidelines for publication of clinical research and a completedCONSORT checklist must be included with all submissions.

Clinical trial registration

N/A

Study protocol

N/A

Data collection

N/A

Outcomes

N/A

Flow Cytometry

Plots

Confirm that:

☒ The axis labels state the marker and fluorochrome used (e.g. CD4-FITC).

☒ The axis scales are clearly visible. Include numbers along axes only for bottom left plot of group (a 'group' is an analysis of identical markers).

☐ All plots are contour plots with outliers or pseudocolor plots.

☒ A numerical value for number of cells or percentage (with statistics) is provided.

Methodology

Sample preparation

CD4+ T cells were purified using the EasySep human CD4+ T-cell isolation kit (STEMCELL Technologies). Flow cytometry was performed to determine the purity of the resulting cells. U2OS cells expressing mGFP, mGFP-Vpr or mGFP-Vpr(1-79) were analyzed to monitor cell cycle progression after propidium iodide staining.

Instrument	BD LSRFortessa
Software	FlowJo
Cell population abundance	10,000 - 20,000 cells were acquired before and after purifying PBMCs from healthy individuals. After purification, CD4+ T cells were more than 90% abundant in three independent experiments as determined by flow cytometry.
Gating strategy	Cells were analyzed by FlowJo software with the gating strategy: FSC vs SSC>>FSC-H vs FSC-A>> SSC-H vs SSC-A>> TCR-b+CD4+. A schematic diagram illustrating gating strategy is provided in the Supplementary Fig. 1.

☒ Tick this box to confirm that a figure exemplifying the gating strategy is provided in the Supplementary Information.
